# Development and application of a novel genome-wide SNP array reveals domestication history in soybean

**DOI:** 10.1038/srep20728

**Published:** 2016-02-09

**Authors:** Jiao Wang, Shanshan Chu, Huairen Zhang, Ying Zhu, Hao Cheng, Deyue Yu

**Affiliations:** 1 National Center for Soybean Improvement, National Key Laboratory of Crop Genetics and Germplasm Enhancement, Nanjing Agricultural University, Nanjing, 210095, China

## Abstract

Domestication of soybeans occurred under the intense human-directed selections aimed at developing high-yielding lines. Tracing the domestication history and identifying the genes underlying soybean domestication require further exploration. Here, we developed a high-throughput NJAU 355 K SoySNP array and used this array to study the genetic variation patterns in 367 soybean accessions, including 105 wild soybeans and 262 cultivated soybeans. The population genetic analysis suggests that cultivated soybeans have tended to originate from northern and central China, from where they spread to other regions, accompanied with a gradual increase in seed weight. Genome-wide scanning for evidence of artificial selection revealed signs of selective sweeps involving genes controlling domestication-related agronomic traits including seed weight. To further identify genomic regions related to seed weight, a genome-wide association study (GWAS) was conducted across multiple environments in wild and cultivated soybeans. As a result, a strong linkage disequilibrium region on chromosome 20 was found to be significantly correlated with seed weight in cultivated soybeans. Collectively, these findings should provide an important basis for genomic-enabled breeding and advance the study of functional genomics in soybean.

Soybean [*Glycine max* (L.) Merr.] seeds are rich in protein and oil and are a major source of nutrients. Cultivated soybeans are known to have been domesticated from their wild annual progenitor (*Glycine soja* Sieb. & Zucc.) in China about 5000 years ago^1^. The proposed centers of domestication for soybean include north-eastern China^2^, the Yellow River valley of northern China^3^, and southern China^4^. In addition, multiple centers of domestication have also been suggested^5^.

Domestication events followed by local adaptations resulted in radical phenotypic transformations from the wild progenitor. The phenotypes of wild soybeans include prostrate plants and small black seeds, while the domesticated landraces are slightly upright plants which have increased seed weights as well as varied seed coat colors. Subsequent to domestication, soybean has continued to be growers’ favorite selection and modern breeding object that is able to produce improved cultivars with erect, high yielding plants that meet human needs^6^.

Modern advancements in gathering genomic information and the development of new genomic analysis methods aid in the ability to link phenotypic variations to genomic differences in crops, including soybean. Two examples of high-throughput platforms for gathering genomic information include next generation sequencing^7^ and the development of DNA microarrays^8^. The development of high-throughput sequencing technologies has allowed researchers to explore how genetic diversity across the whole genome of soybeans was shaped by domestication by comparing the patterns of genetic variation between wild and cultivated soybeans^6^,^9^,^10^. Recently, 302 wild, landrace, and improved soybean accessions were sequenced, and several genes related to the domestication and improvement of soybeans were identified, providing resources for genomics-enabled improvements in soybean breeding^10^.

Compared to a whole genome sequencing approach, an array approach is a faster, cheaper, and more convenient alternative genotyping technology for germplasm screening^11^,^12^. In soybean, 1.5 and 6 K single nucleotide polymorphism (SNP) arrays have been developed for quantitative trait locus (QTL) mapping and association studies^13^,^14^. In addition, an Illumina Infinium BeadArray, SoySNP50K array is also available and has been used to screen the entire USDA Soybean Germplasms Collection^15^,^16^. Recently, a 180 K AXIOM^®^ SoyaSNP array was developed and validated in 222 diverse soybean lines^17^. However, higher-resolution arrays are needed for large scale genomic level genotyping in soybean to investigate domestication history and ultimately provide information and genetic resources for future breeding.

Here we report the development of the NJAU 355 K SoySNP array that contains 355,595 SNPs covering the whole soybean genome. This high-density SNP array was used to track the evolutionary history of soybeans and detect the genetic bases for soybean breeding in the genomes of 105 wild and 262 cultivated soybean accessions. A genome-wide scan of artificial selection signals was also conducted to detect candidate genes related to agronomically important traits. Among these traits, seed weight was taken as an example in further genome-wide association study (GWAS) to identify significantly related loci. Additionally, the high-resolution NJAU 355 K SoySNP array developed in our study provides a valuable resource for genetic diversity analysis and the investigation of agronomically important genes in soybean.

## Results

### Developing a high-density, genome-wide SNP array (NJAU 355 K SoySNP array)

**Figure 1:**
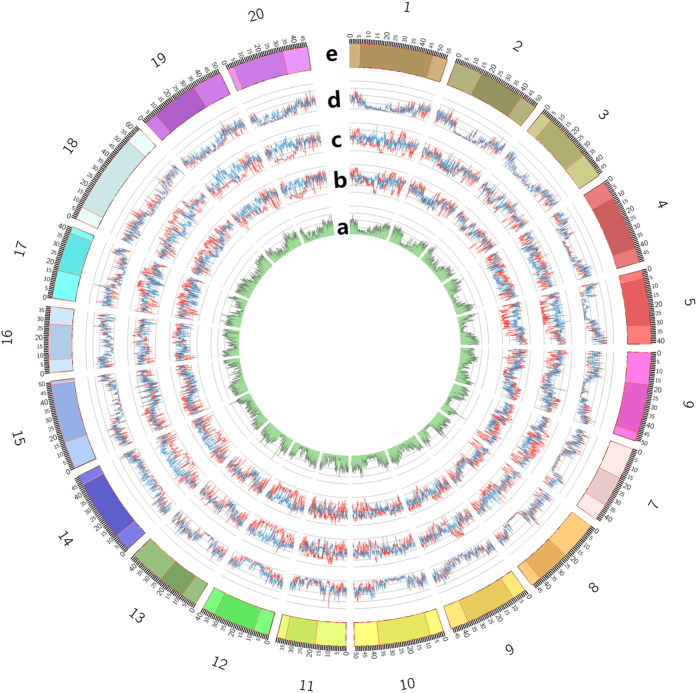
Polymorphism comparisons between wild and cultivated soybeans. (**a**) Distribution of SNP number in the 100-kb sliding windows. (**b**) Tajima’s D value in the 100-kb sliding windows of wild (blue line) and cultivated (red line) soybeans. (**c**) Minor allele frequency (MAF) value in the 100-kb sliding windows of wild (blue line) and cultivated (red line) soybeans. (**d**) Genetic diversity (π) value in the 100-kb sliding windows of wild (blue line) and cultivated (red line) soybeans. (**e**) Chromosome structure with centromeric regions in a darker color and pericentromeric regions in a lighter color (scale is in Mb).

An Affymetrix Axiom Genome-Wide BOS 1 Array containing 355,595 SNPs (NJAU 355 K SoySNP array) was developed and used to genotype 122 wild and 272 cultivated samples. Twenty-seven of the 394 samples were excluded due to the low sample quality control rates and the remaining 367 samples (105 wild and 262 cultivated soybeans) were included in the subsequent study. The 355,595 SNPs that were identified in the remaining 367 individuals were classified based on SNP quality control metrics into six categories (http://www.affymetrix.com/) (Supplementary Fig. S1). Approximately 18% of the SNPs, classified as ‘Other’, were filtered out based on the low quality genotypes below the acceptable threshold level. The remaining 292,053 high-quality SNP markers were used for further genetic analysis. The uneven distribution of SNP markers on chromosomes has been shown in Fig. 1. Of the 292,053 SNPs, 291,962 SNPs were mapped to chromosomes and 91 SNPs were located in unanchored sequence scaffolds. Of the 291,962 SNPs, 115,245 (39.47%) SNPs were located in pericentromeric regions and 176,717 (60.53%) were targeted to the arms of the 20 soybean chromosomes. The pericentromeric regions are recombination-suppressed genomic blocks that are rich with transposable elements. SNP identification is difficult in these regions. Therefore, the identified markers were sparse in these regions. Of the 291,962 chromosomal SNPs, the average SNP spacing was approximately 3.3 kb along the 20 chromosomes of soybean (975 Mb). The inter-SNP spacings of 93.6% of the SNPs were shorter than 9 kb. Only 66 SNPs had a marker spacing of >100 kb (Supplementary Fig. S2). A total of 90,088 (30.86%) of the 291,962 chromosomal SNPs resided in genes, of which 25,336 SNPs were located in coding DNA sequences, 48,993 SNPs in introns, 6,885 SNPs in 5′-untranslated regions (UTRs), and 8,874 SNPs in 3′-UTRs (Supplementary Table S1). Additionally, 4.56% of the total SNPs were non-synonymous sites and 3.94% were synonymous sites. We identified 13,444 non-synonymous SNPs in 11,407 genes, including 994 nonsense SNPs in 977 genes that resulted in start codon changes, premature stop codons, or elongation of the transcripts. This SNP data set provided us with a new resource for soybean biology and breeding. A total of 37,370 genes were covered by high quality SNP markers, accounting for 68.98% of the 54,175 predicted genes annotated in the version of Glyma.Wm82.a1.v1.1 (www.phytozome.net). The values of minor allele frequencies (MAF) were calculated for each SNP marker in the 367 samples. We found that 215,657 of the 292,053 SNPs (73.84%) had MAF values greater than 10% among the soybean accessions, making them potentially useful for further crop breeding applications such as GWAS^19^.

### Polymorphisms comparison between wild and cultivated soybean accessions

To compare the genetic diversity between wild and cultivated soybeans, we calculated the average pairwise SNP numbers of both wild and cultivated soybeans. Average pairwise SNP numbers were found to be lower in the cultivated soybean population compared to those of wild soybeans (Supplementary Fig. S3). Among all of the SNPs that we studied, a large proportion (82.73%) of the SNPs were detected in both wild and cultivated soybeans, suggesting that most of the polymorphism observed in cultivated soybeans came from their wild progenitors. There were 13,757 SNPs (4.71%) unique to the *G. soja* accessions and 4,339 (1.49%) unique to the *G. max* lines. The 13,757 loci that were polymorphic in *G. soja*, but monomorphic in *G. max*, suggest that these loci were subject to artificial selection. Additionally, 32,347 (11.08%) SNPs showed no polymorphism (Supplementary Table S2). For further estimation, π, a statistic used to measure the genetic diversity in a population, was calculated using a sliding window analysis (Fig. 1). The π value of wild soybeans was significantly higher than that of cultivated soybeans (two-tailed t-test, *P* = 1.92 × 10^−28^), indicating that wild soybeans maintained a higher level of nucleotide diversity. These results add the evidence that wild soybeans should be preserved for the further exploitation of valuable genetic resources^20^.

We also measured the MAF values using a sliding window analysis (Fig. 1). The results showed that more alleles with minor frequency are detected in cultivated soybeans. Meanwhile, Tajima’s D (Fig. 1), which was used to determine allele frequency change was compared between wild and cultivated soybeans. The values of Tajima’s D in cultivated soybeans were lower than those in the wild samples. These results combined suggest that there was a recent selective sweep in cultivated soybeans.

**Figure 2:**
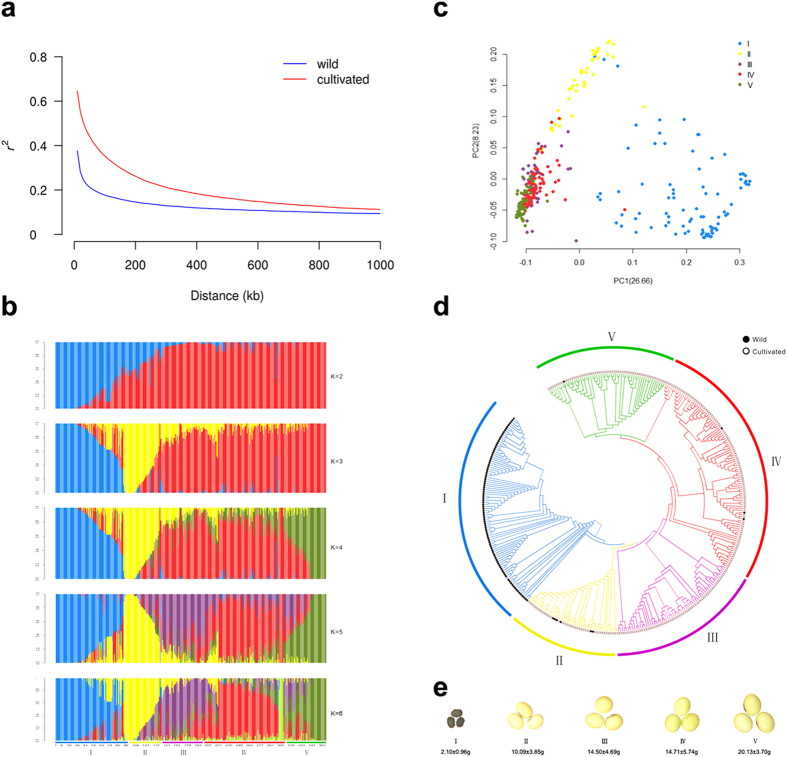
The population structure of 367 soybean accessions and LD decay of wild and cultivated soybeans. (**a**) LD decay estimated by the squared allele frequency correlation (*r*^*2*^) against the distance between polymorphic sites in wild (blue) and cultivated (red) soybeans. (**b**) Population structure analysis of 367 accessions using ADMIXTURE. Each color represents a single population. Each vertical column represents one accession and each colored segment in each column represents the proportion contributed from ancestral populations. The numbers of clusters (K) were set from 2–6. The 367 accessions were divided into 5 groups (I–V). (**c**) PCA of the 367 accessions. Individuals from the same group are represented by the same color. (**d**) A neighbor-joining tree of the 367 accessions. (**e**) Variation in seed weight in soybeans. Typical seeds of each group were shown.

Linkage disequilibrium (LD) analysis was also performed in both wild and cultivated soybeans. The distance over which LD decays to half of its maximum value was 80 kb in wild soybeans and 130 kb in cultivated soybeans (Fig. 2a). The higher LD value in cultivated soybeans is consistent with the finding that the effect of artificial selection exists in this population.

### A population structure analysis reveals eco-geographic and seed weight distribution patterns in wild and cultivated soybeans

To examine the population structure and relatedness among soybeans, we performed population clustering with ADMIXTURE and constructed a neighbor-joining tree. In the ADMIXTURE (Fig. 2b), when K = 2, a primary division existed between wild and cultivated soybeans. And when K = 3, a new group appeared admixed with wild and cultivated soybeans. At higher levels of K, new groups emerged within cultivated soybeans. When K = 5, cultivated soybeans clustered into three major groups and no apparent new group appeared when K = 6. These results were further supported by principal component analysis (PCA) (Fig. 2c) and phylogenetic analysis (Fig. 2d). According to both population structure and the phylogenetic analysis, the accessions were partitioned into five groups (I to V). All individuals in group I were wild soybeans except for one cultivated soybean sample. Group II was admixed, with six wild and forty cultivated soybeans, and groups III through V mostly contained cultivated soybeans.

Based on the climatic and geographical condition, planting time, and cropping systems of soybeans, the habitat of soybean is divided into the northern region (NR), the Huang-Huai region (HR), and the southern region (SR) (Supplementary Fig. S4)^21^. Eco-geographical distributions were summarized for each group (Supplementary Table S3). Group I was slightly biased towards the NR. Group II mainly centered in the HR and the NR. Group III was evenly dispersed across China, and Group IV and V mainly from the SR. The results suggest that cultivated soybeans probably originated from the northern and middle regions of China, from where it spread to other regions. Furthermore, the eco-geographical distributions were identified for wild, landrace, and improved soybeans respectively (Table 1). Most wild accessions from NR, HR and SR were gathered in group I. For landraces, all groups except group I contained accessions from the three ecological regions. For improved soybeans, only group III possessed accessions from all three ecological regions, suggesting that improved accessions in group III are distributed widely in China.

Seed weight is an important target trait that was selected during soybean domestication. In our study, the averaged values of 100-seed weight increased from group I to V (from 2.10–20.13 g) (Supplementary Table S4; Fig. 2e). This trend was also observed in wild, landrace, and improved soybeans separately. For instance, the values increased from 10.59 g in group II to 19.77 g in group V for landraces and 14.42 g in group II to 23.71 g in group V for improved soybeans (Supplementary Table S4). These results support that seed weight increased gradually after domestication from wild soybean.

### Identification of signs of artificial selection in domestication

**Figure 3:**
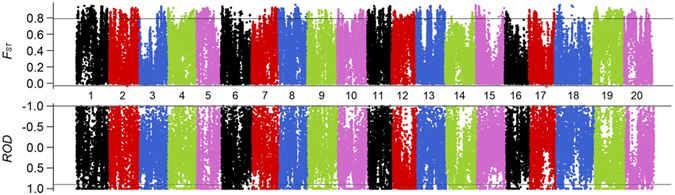
Artificial selection signal detection between cultivated and wild soybeans. A genome-wide view in a 10-kb sliding window of *F*_ST_ and *ROD*. The lines represent the 95% tails for the empirical distribution of *F*_ST_ and *ROD* statistics, respectively.

To characterize domestication of soybean at the genomic level, we identified genes that were subject to artificial selection by comparing polymorphism levels (*ROD* = 1−π_cul_/π_wild_) and genetic differentiation (*F*_ST_) between the cultivated and wild species (Fig. 3). The genome-wide scan revealed that 1,614 non-overlapping windows appeared to have been affected by selection during domestication. These selection windows accounted for only 1.66% of the whole genome (975 Mb) and were not evenly distributed throughout the genome. Instead, some of these windows clustered together with lengths ranging from 20–220 kb, which might be a result of genetic linage to one or more loci affecting an important agronomic trait. Altogether 1,128 genes exist in the selective sweep regions and account for 2.08% of the 54,175 predicted genes in the cultivated soybean genome. To annotate these candidate genes, BLASTp searches were performed in *Arabidopsis* and rice functional gene databases^22^,^23^. The results revealed that some of these genes were involved in domestication-related agronomic traits such as stress response, seed weight, seed composition, plant height and flowering time (Supplementary Table S5).

Increased seed weight is a major trait associated with the domestication of soybeans^6^. Among the candidate domestication genes, 16 genes were found to be associated with seed weight. We then compared the location of these genes with mapped seed weight related QTLs reported in Soybase (http://soybase.org/). The results showed that six of these genes had overlapping regions with mapped seed weight related QTLs (Supplementary Table S5). For example, *Glyma06g 12800*, a homologous gene of *GS5,* which encodes a putative serine carboxypeptidase positively regulating grain size, genetically maps to two QTLs related to seed weight (sw 29–3 and sw 30–1) in rice^24^. Furthermore, three non-synonymous substitutions with significant differences of allele frequency between wild and cultivated soybeans were detected among these six genes. For instance, *Glyma01g41990* is an ortholog of rice GIF1 (Grain Incomplete Filling 1), an enzyme involved in the carbohydrate metabolism pathway that controls seed weight during grain development^25^. We detected one non-synonymous substitution (T to C) in this gene which resulted in the V326A amino acid mutation. In wild soybeans only 20.48% of the individuals maintained the amino acid V, while in cultivated soybeans almost all of the individuals (96.18%) maintained the amino acid V. This mutation, located in the glycosyl hydrolases family 32 N-terminal domain of GIF1, may affect gene function.

### Detection of seed weight candidate loci by GWAS

**Figure 4:**
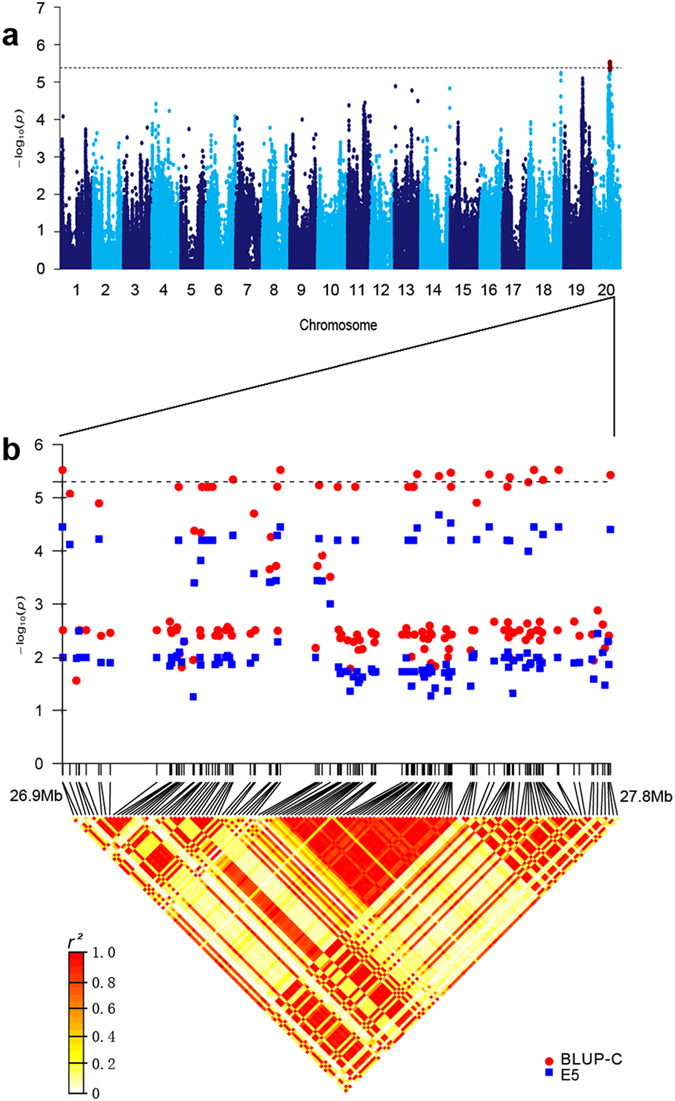
GWAS results for seed weight in cultivated soybeans. (**a**) Manhattan plot for seed weight in E5 environment. The dashed horizontal line depicts a significant threshold level (4.82 × 10^−6^). The twelve significant SNPs are indicated with red dots, and the candidate genes are listed in Table 2. (**b**) A strong regional linkage disequilibrium was observed on chromosome 20 from 26.9–27.8 Mb.

The 100-seed weight of the wild soybeans in four environments and cultivated soybeans in nine environments were assayed and showed that there were broad variations in seed weight for either cultivated soybeans or wild soybeans (Supplementary Fig. S5; Table S6 and S7). We found that heritability is high for both cultivated and wild soybeans. Most of the signals identified by GWAS were in regions previously reported to contain seed-weight QTLs (Table 2). Thirteen significant SNPs were detected in cultivated soybeans. One of these 13 SNPs, located on chromosome 11, was observed in two environments and showed a significant marker-trait association, with a *P*-value as low as 2.479 × 10^−6^ (Supplementary Fig. S6b & d; Table 2). There were 17 genes detected in the 130 kb flanking region (LD decay distance of cultivated soybeans) of this SNP. Among these genes, *Glyma11g03360* is homologous to *Os04g33740* (*GIF1*) that regulates grain filling and size in rice^25^, and *Glyma11g03430* is homologous to *Os11g12740* (*sp1*), a gene that participates in panicle elongation and grain size^26^. Thus, these two genes in soybean could be considered as candidate genes for seed weight. The other 12 SNPs were detected in the E5 environment under the threshold of *P* < 4.82 × 10^−6^, and were organized as a cluster on chromosome 20 (26.9–27.8 Mb) (Fig. 4). Under the threshold of *P *< 1 × 10^−4^, all these 12 SNPs were repetitively detected in the best linear unbiased prediction (BLUP) data set. Strong and extensive linkage disequilibrium was observed in this region, an indication of human selection. Furthermore, considering the LD decay distance, we extended this region from 26.8–27.9 Mb. QTLs associated with seed weight have been reported multiple times in this region (Table 2).

In addition, we performed GWAS in wild soybeans and identified 12 SNPs significantly related to seed weight, which are located on chromosomes 11, 13, and 18 (Supplementary Fig. S7; Table 2). These SNPs are anchored to nine regions (Table 2). Within these regions, three genes, *Glyma11g05760*, *Glyma18g05240*, and *Glyma18g43500*, are considered to be candidate genes for seed weight, since their homologous genes (*SWN, IKU2* in *Arabidopsis,* and *LRK1* in rice, respectively) participate in the regulation of seed size or grain weight^27^,^28^,^29^.

## Discussion

Our results support that cultivated soybeans were gradually domesticated from wild soybeans^1^ and that cultivated soybeans probably originated from the northern and middle regions of China and then spread to other regions. This process is confirmed by the fact that Chinese civilization originated from Yellow River Valley, and then spread across China^30^. Domestication of soybean in other regions such as south China has also been proposed by Guo *et al.*^31^. Two main reasons could account for the different conclusions. Firstly, ~300K high-quality SNPs covering the whole genome were used in our genotyping analysis, whereas only 56 genome-wide distributed microsatellites were used in the study of Guo *et al.*^31^. Secondly, 367 accessions including 105 wild and 262 cultivated accessions covering all three ecological habitats in China and representing the full geographic range of wild and cultivated soybeans were used in our phylogenetic analysis. In contrast, 231 wild soybeans and 79 landrace soybeans from the natural distribution areas in East Asia were collected by Guo *et al.*^31^. More extensive studies are still needed to shed light on the history of soybean domestication.

As soybean is a photoperiod and temperature sensitive crop, it is difficult for specific soybean accessions to adapt to various environments^32^. Hence, the discovery of widely adaptive accessions from the breeding process will be of great value. In our study, group III was the only one that contained improved soybean lines from all three regions (Table 1) and our phylogenetic tree analysis supports that there might be a common ancestor of improved accessions in group III. The wide distribution of improved accessions in group III suggests that the ancestors of this group had the potential to adapt to different ecological regions. The accessions in group III could be applied to overcome the environmental sensitivity, break planting boundaries, and breed varieties adapted to different ecological regions. In the breeding process, accessions in group III could be collected and planted in different ecological regions. Subsequently, the accessions that can survive in various ecological regions could be selected as the backbone parents to cultivate widely adaptive varieties.

Seed weight is a vital agronomical trait in soybean domestication and breeding. In this study, the regions related to seed weight identified by GWAS in cultivars and wild soybeans do not overlap with each other. The possible explanation could be that seed weight is a complicated trait controlled by multiple genes, the genes controlling seed weight in wild soybeans may be different from those in cultivated soybeans^33^. Besides, wild soybeans are highly influenced by natural selection, while cultivated soybeans are subjected to artificial selection, thus loci under natural selection could be different from those under artificial selection, which is a more conscious and directional selection. In addition, the loci associated with seed weight in wild soybeans in our study did not overlap with the loci identified in wild soybeans by some other researchers such as Li *et al.*^34^. The inconsistency could be caused by the following reasons. On one hand, different mapping populations were used. We used 105 wild accessions, which represent the diversity of natural *G. soja* population covering three ecological habitats in China. In contrast, Li *et al.*^34^ used two pedigree populations (BC_2_F_4_ populations) produced by inter-species crosses. On the other hand, we used association mapping, which explored all of the recombination events that have occurred during the evolution process, while Li *et al.*^34^ used a linkage analysis method on pedigree populations. These inconsistencies suggest that the pool of seed weight controlling loci is abundant and potentially provide new resources for the improvement of cultivated soybean.

Genome-wide SNP arrays have been developed and widely applied in GWAS in plants^16^,^35^,^36^,^37^,^38^. For example, in maize, a Maize SNP50 array containing 56,110 SNP markers was used in GWAS and led to the identification of promising candidate loci associated with resistance to northern corn leaf blight^35^. In rice, a RiceSNP50 array was used in the GWAS and identified loci controlling the ratio of length/width of rice grains^37^. In soybean, a SoySNP50 K array was used to conduct GWAS and identified QTLs controlling seed protein and oil content^16^. Notably, a high-throughput 180 K AXIOM^®^ SoyaSNP array of which 83.8% of SNPs had an inter-marker spacing shorter than 9 kb has been developed recently, which is probably suitable for GWAS in soybean^17^. Here, we developed a higher-density NJAU 355 K SoySNP array of which 95.2% of SNPs had an inter-marker spacing shorter than 9 kb (Supplementary Fig. S2). The high-density array would be a powerful tool for genome-wide association studies in soybean breeding.

In summary, the NJAU 355 K SoySNP array was used to genotype 367 wild and cultivated soybean accessions. With the genotyping results, we conducted a population genetic analysis to reveal the domestication history of soybeans. Further, tracing the domestication footprint in combination with phenotypically identifying causative variations will inform the future detection of agronomically important loci. In addition, a set of SNPs with high breeding application values can be used to construct medium- or low-density SNP arrays for specific germplasm screening and identification for molecular breeding. The data generated in this study provide a valuable resource for further soybean genomic breeding.

## Materials and Methods

### Pipeline of NJAU 355 K SoySNP Array Design

To design the assay (Affymetrix Axiom Genome-Wide BOS 1 Array), data from 31 re-sequenced soybeans (including 17 wild and 14 cultivated soybeans)^9^ and wild soybean IT182932^18^ were used (Supplementary Fig. S8). The SNPs with minor allele frequency (MAF) ≥0.10 and missing genotype >0.2 were eliminated. 16 bp sequences on either side of the SNPs were used to do BLAST searches on the Williams 82 reference genome. The sequences with multiple hits were eliminated. The reference sequence version used in this study was Glyma.Wm82.a1.v1.1 (www.phytozome.net). 1,095,943 SNPs were detected from both the 31 re-sequenced samples and one wild soybean (Supplementary Fig. S8). Given the high LD in soybeans, we used Haploview to identify the tag SNPs with a threshold correlation coefficient (r^2^) < 0.8^39^. Next, we performed a 1,680 bp sliding window analysis according to the principle of even distribution. If a window contained tagSNP loci, we did not introduce new SNPs. Otherwise, genic SNPs rather than intergenic SNPs will be introduced to the window containing no tagSNP loci. Finally, a set of VIP SNPs was supplemented, with 60,800 SNPs in SoySNP50 K^15^, 2,103 SNPs from Soybase and 3,671 SNPs from our lab. After filtering by Affymetrix, there were 355,595 SNPs represented by 609,883 probe sets. Each of the 254,288 SNPs was represented by two probe sets, and each of the 101,307 SNPs was represented by one probe set. These 355,595 SNPs were used to produce the NJAU 355 K SoySNP array. In addition, 4,000 dish quality control probes designed from sequences with no variation were used for sample quality control.

### SNP genotyping

Genotyping and QC procedures for markers and samples were conducted according the Axiom^®^ Genotyping Solution Data Analysis User Guide and its Best Practice Supplement (http://www.affymetrix.com/). In brief, we first performed sample quality control based on both the values of Dish Quality Control (DQC) and sample call rate using the Affymetrix^®^ Power Tools (APT) software package, version 1.15.0. The DQC is the primary chip-level quality metric for Axiom microarrays and measures signal contrast distributions of probes that complement genomic sequence with no expected polymorphism. The value of sample call rate refers to the ratio of genotype-called SNPs to attempted SNPs in a sample, which is mainly used for identifying contaminated samples. All samples in our study had a DQC value >0.82, and 27 samples with a call rate <97% were excluded from further genotyping analysis. The remaining 367 samples were genotyped again using APT and the standard genotyping output files were then post-processed in the SNPolisher version 1.3.6.7, an R package specifically designed by Affymetrix to filter out SNPs with low quality. Its Ps_Classification function classifies SNPs/probe sets based on SNP QC metrics into six categories including ‘PolyHighResolution’, ‘MonoHighResolution’, ‘Off-Target Variant’, ‘CallRateBelow-Threshold’, ‘NoMinorHom’ and ‘Other’. Finally, all 63,447 SNPs in the ‘Other’ category and 95 insertion/deletion SNPs were excluded. 292,053 SNPs were retained for further analysis. The number of SNPs is estimated to provide approximately one SNP per 3.3 kb along the 20 chromosomes of soybean. Annotation of the remaining SNPs was performed using SnpEff (version 3.6c)^40^, referring to soybean genome annotation (Glyma.Wm82.a1.v1.1) downloaded from the Phytozome database (http://www.phytozome.net/).

### Soybean materials and phenotypic data collection

A total of 394 accessions including 122 wild and 272 cultivated accessions covered all three ecological habitats in China (NR, HR and SR)^21^, which were divided approximately by the Yellow River and the Yangtse River (Supplementary Fig. S4). These accessions were provided by the Germplasm Storage of Chinese National Center for Soybean Improvement (Nanjing Agricultural University, Nanjing, China). All 394 accessions were genotyped, but only the 367 samples that passed a DQC threshold of 0.82 and a call rate of 97% were included for further analysis. We performed polymorphism detection of the 367 accessions (Supplementary Table S8), including 105 wild and 262 cultivated accessions. Among the cultivated accessions, there were 197 landraces, 55 improved accessions and 10 accessions with unknown evolution type. Young leaves were collected and quickly frozen in liquid nitrogen. Total DNA was extracted with the cetyltrimethyl ammonium bromide (CTAB) method (OD 260/280: 1.7–2.1, ≥5 μg)^41^.

A subset of 211 accessions from 262 cultivated soybeans was used for association analysis, and these accessions were grown from 2011–2013 in Nanjing, Nantong, and Yangzhou, China. Each location within each certain year is considered to be a single environment; thus, this study contained nine environments, which were designated as E1 to E9 (E1: Jiangpu_2011, E2: Jiangpu_2012, E3: Jiangpu_2013, E4: Nantong_2011, E5: Nantong_2012 and E6: Nantong_2013, E7: Yangzhou_2011, E8: Yangzhou_2012, E9: Yangzhou_2013). All accessions were arranged in a completely randomized block design with three replicates in each test location. For each accession, the replication of 80 plants was arranged in each of a four square meter matrix plot in the field (four rows, ten hills for each row with 20 cm space, and thinning to two plants left for each hill after two weeks). After maturity, four individuals for each accession were randomly screened for 100-seed weight measurement with four technical repeats.

Ninety-six wild accessions from 105 wild soybeans formed the wild association panel. Phenotype investigation was performed in 2011 in Nanyang and from 2011–2013 in Nanjing, designated as e1–e4 (e1: Nanyang _2011, e2: Jiangpu_2011, e3: Jiangpu_2012, e4: Jiangpu_2013). The experimental design was the same as for the cultivated soybeans. For each accession, 20 plants in four hills were arranged in four square meter plots (each hills with five individuals). All plants of each accession in each replicate were used to measure 100-seed weight with four technical repeats. All field management requirements during the growing period, including watering, weed management, and fertilization, were performed equally in each test location.

Phenotype data were analyzed using R software (http://www.R-project.org/). A best linear unbiased prediction (BLUP) model in R (lme4 package) was fitted to account for the year, trial and location effects together with their interactions. The intercept value from the BLUP model was the random effect of each variety representing the breeding value of each variety. The breeding values from the BLUP model consisted of a new phenotype data for GWAS, defined as BLUP-C and BLUP-W for cultivated and wild soybeans respectively.

### Polymorphism parameters estimates

We separated our samples into wild and cultivated populations. Then we used VCFtools V0.1.12b^42^ to calculate genetic diversity (π), MAF, and Tajima’s D values of wild and cultivated population by applying a 100 kb non-overlapping sliding window over the whole genome for the two populations. The plotted parameters were displayed by using Circos 0.66 (http://circos.ca).

TASSEL 5^43^ was used to perform LD analysis of wild and cultivated soybeans by estimating the squared allele frequency correlation (r^2^) of alleles using all SNPs. The distance that the LD decays to half of its maximum value was calculated. The LD decay graphs of both wild and cultivated soybeans were plotted using an R script.

### Population structure analysis

We used PLINK V1.07^44^ to perform SNP filtering by setting MAF to 0.05 and a call rate to 0.1. The pruned data contained 224,774 SNPs, which were used to construct population structure by using ADMIXTURE^45^ for maximum likelihood estimation. The number of clusters (K) was set from 2–6. PCA analysis was performed using the pruned data mentioned before and generated a pairwise identity-by-state (IBS) distance matrix of 367 individuals. Population stratification was distributed by a multidimensional scaling plot.

### Construction of a phylogeny tree

We first used SNPhylo^46^ to perform SNP filtering by setting MAF to 0.05 and a call rate to 0.1. Then we used the rest of the 224,774 SNPs to construct a neighbor-joining tree with 1,000 bootstrap steps, calculated and distributed by MEGA6^47^.

### Detection of artificial selection regions and genes

A composite scoring system was used to determine whether a 10 kb sliding window was under selection. This approach is similar to those applied for rice and common bean where a reduction in nucleotide diversity and *F*_ST_ was applied to discover domestication-related genes^48^,^49^. Here a 10-kb window was considered as a domestication selection window if it was in the upper 95% of the pool’s empirical distribution for both *ROD* values (estimated as 1−π_cul_/π_wild_) and *F*_ST_ statistics. The genes located in these selection windows were chosen as candidate genes. To annotate the candidate genes, we performed BLASTp searches in *Arabidopsis* and the rice functional gene database using amino acid sequences of these genes with an E-value cutoff of 1e^−20 ^22,23.

### Genome-wide association study (GWAS)

After excluding SNPs with a MAF <0.05, 207,608 SNPs were left for cultivated soybeans and 239,658 SNPs for wild soybeans. GWAS was performed by using a compressed mixed linear model^50^. All analyses were conducted with the GAPIT package^51^. The population structure was accounted for by PCA and the relatedness was calculated by VanRaden method^52^. The first five principles of PCA were used as a covariate matrix for compressed mixed model for both cultivated and wild soybeans. The threshold for significant association was set to 1/n (n is the marker numbers for each association panel, for cultivated soybeans, *P* < 4.82 × 10^−6^, for wild soybeans *P* < 4.17 × 10^−6^)^53^.

To uncover the candidate genes underlying association signals, we first selected the genes tagged by the most significant SNPs and the nearby genes within 130 kb upstream and downstream in cultivated soybeans and 80 kb in wild soybeans. The nearby distance of the lead SNP was based on LD decay calculated previously. Then we annotated the significant genes identified by GWAS using the method described above. The genes related to seed weight were selected as candidate genes.

## Additional Information

**How to cite this article**: Wang, J. *et al.* Development and application of a novel genome-wide SNP array reveals domestication history in soybean. *Sci. Rep.* **6**, 20728; doi: 10.1038/srep20728 (2016).

## Supplementary Material

Supplementary Information

## Figures and Tables

**Table 1:** Summary of ecological distribution of accessions of different evolutionary types from different groups.

Evolutionary Types	Ecological Regions	Number of Accessions	I	II	III	IV	V
Wild	NR^a^	26	0.962	0.038	0	0	0
	HR^b^	30	0.867	0.133	0	0	0
	SR^c^	46	0.913	0.022	0	0.043	0.022
Landrace	NR	16	0	0.438	0.063	0.250	0.250
	HR	43	0.023	0.419	0.116	0.395	0.047
	SR	118	0	0.034	0.229	0.602	0.136
Improved	NR	5	0	0.200	0.800	0	0
	HR	14	0	0.143	0.357	0	0.500
	SR	29	0	0	0.138	0.069	0.793

^a^Northern region.

^b^Huang-Huai region.

^c^Southern region.

**Table 2:** Details of loci associated with seed weight identified via a genome-wide association study (GWAS) in soybean.

Pop^a^	Chr.^b^	MSS^c^ position	MSS *P* value	No. of SNP^d^	Significant region^e^		Total SNPs^f^	Candidate genes^g^	QTLs	Env.^h^
					Start	End				
C^i^	11	2338406	2.479E-06	1	2211407	2464139	109	*Glyma11g03360* (*GIF1*^25^)*; Glyma11g03430 (sp1*^26^)	sw^k^ 37−9^54^; sw 6−3^55^	E1,E3
C	20	27308646	2.999E-06	12	26835715	27938298	314		sw 34−5^56^; sw 35−5^56^; sy^l^ 9−1^57^; sw 9−1^57^; sy 14−1^58^	E5
W^j^	11	4135246	4.053E-07	3	4054184	4250040	90	*Glyma11g05760* (*SWN*^27^)	sw 37−9^54^	BLUP-W,e2
W	11	12287129	2.018E-06	1	12217654	12366256	31		sw 20−1^59^ ; sw 20−4^59^; sw 20−3^59^	e3
	e3									
W	11	14211486	1.129E-06	1	14133222	14289334	22		sw 20−1^59^; sw 20−4^59^; sw 20−3^59^; sw 32−1^60^;sw 11−1^61^; sw 4−1^62^	e3
W	11	34177488	4.024E-07	1	34097703	34250397	20		sw 32−11^60^; sw 36−11^56^; sw 35−9^56^	e4
	e4									
W	13	10352822	3.055E-06	1	10298834	10431757	36		sy 18−3^61^	e2
W	13	28066134	2.819E-06	1	27986488	28146130	76			BLUP-W
W	13	35835159	1.711E-06	1	35755522	35914739	96		sy 5−3^55^	e4
W	18	3968571	2.864E-06	1	3889812	4038596	84	*Glyma18g05240* (*IKU2*^28^)		e1
W	18	53103514	7.827E-08	2	52880506	53182807	136	*Glyma18g43500 (LRK1*^29^)	sw 37−4^54^; sw 4−2^62^; sw 10−9^63^	BLUP-W,e3

^a^Population.

^b^Chromosome.

^c^Most significant SNP.

^d^Number of significant SNPs.

^e^Significant region was defined as the upstream and downstream of LD decay distance flanking the significant SNPs.

^f^Total number of SNPs in significant regions.

^g^Candidate genes refer to the soybean genes in significant region which is homologous to seed weight related genes in rice or *Arabidopsis*, shown in parentheses.

^h^Environments.

^i^Cultivated soybeans.

^j^Wild soybean.

^k^Seed weight.

^l^Seed yield.

## References

[b1] Hymowitz T. Speciation and Cytogenetics. In Soybean: improvement, production, and uses (eds Boerma H. R. & Specht J. E. ) 97–136 (Am. Soc. of Agro, 2004).

[b2] Fukuda Y. Cytogenetical studies on the wild and cultivated manchurian soybeans (*Glycine L.*). Jpn. J. Bot. 6, 489–506 (1933).

[b3] Li Y. H. *et al.* Genetic diversity in domesticated soybean (*Glycine max*) and its wild progenitor (*Glycine soja*) for simple sequence repeat and single-nucleotide polymorphism loci. New Phytol. 188, 242–253 (2010). 20618914 10.1111/j.1469-8137.2010.03344.x

[b4] Gai J. Y. *et al.* Studies on the evolutionary relationship among eco-types of *G. max* and *G. soja* in China. Acta. Agron. Sin. 26, 513–520 (2000).

[b5] Xu H., Abe J., Gai Y. & Shimamoto Y. Diversity of chloroplast DNA SSRs in wild and cultivated soybeans: evidence for multiple origins of cultivated soybean. Theor. Appl. Genet. 105, 645–653 (2002). 12582476 10.1007/s00122-002-0972-7

[b6] Li Y. H. *et al.* Molecular footprints of domestication and improvement in soybean revealed by whole genome re-sequencing. BMC Genomics 14, 579 (2013). 23984715 10.1186/1471-2164-14-579 PMC3844514

[b7] Davey J. W. *et al.* Genome-wide genetic marker discovery and genotyping using next-generation sequencing. Nat. Rev. Genet. 12, 499–510 (2011). 21681211 10.1038/nrg3012

[b8] Gupta P. K., Rustgi S. & Mir R. R. Array-based high-throughput DNA markers for crop improvement. Heredity 101, 5–18 (2008). 18461083 10.1038/hdy.2008.35

[b9] Lam H. M. *et al.* Resequencing of 31 wild and cultivated soybean genomes identifies patterns of genetic diversity and selection. Nat. Genet. 42, 1053–1059 (2010). 21076406 10.1038/ng.715

[b10] Zhou Z. *et al.* Resequencing 302 wild and cultivated accessions identifies genes related to domestication and improvement in soybean. Nat. Biotechnol. 33, 408–414 (2015). 25643055 10.1038/nbt.3096

[b11] Viquez-Zamora M. *et al.* Tomato breeding in the genomics era: insights from a SNP array. BMC Genomics 14, 354 (2013). 23711327 10.1186/1471-2164-14-354 PMC3680325

[b12] Yu H., Xie W., Li J., Zhou F. & Zhang Q. A whole-genome SNP array (RICE6K) for genomic breeding in rice. Plant Biotechnol. J. 12, 28–37 (2014). 24034357 10.1111/pbi.12113

[b13] Hao D. *et al.* Identification of single nucleotide polymorphisms and haplotypes associated with yield and yield components in soybean (*Glycine max*) landraces across multiple environments. Theor. Appl. Genet. 124, 447–458 (2012). 21997761 10.1007/s00122-011-1719-0

[b14] Akond M. *et al.* A SNP-based genetic linkage map of soybean using the SoySNP6K Illumina Infinium BeadChip genotyping array. J. Plant Genome Sci. 1, 80–89 (2013).

[b15] Song Q. *et al.* Development and evaluation of SoySNP50K, a high-density genotyping array for soybean. PLoS One 8, e54985 (2013). 23372807 10.1371/journal.pone.0054985 PMC3555945

[b16] Hwang E. Y. *et al.* A genome-wide association study of seed protein and oil content in soybean. BMC Genomics 15, 1 (2014). 24382143 10.1186/1471-2164-15-1 PMC3890527

[b17] Lee Y. G. *et al.* Development, validation and genetic analysis of a large soybean SNP genotyping array. Plant J. 81, 625–636 (2015). 25641104 10.1111/tpj.12755

[b18] Kim M. Y. *et al.* Whole-genome sequencing and intensive analysis of the undomesticated soybean (*Glycine soja* Sieb. and Zucc.) genome. Proc. Natl. Acad. Sci. USA 107, 22032–22037 (2010). 21131573 10.1073/pnas.1009526107 PMC3009785

[b19] Tabangin M. E., Woo J. G. & Martin L. J. The effect of minor allele frequency on the likelihood of obtaining false positives. BMC Proc. 3 Suppl 7, S41 (2009). 20018033 10.1186/1753-6561-3-S7-S41 PMC2795940

[b20] Li Y. H. *et al.* De novo assembly of soybean wild relatives for pan-genome analysis of diversity and agronomic traits. Nat. Biotechnol. 32, 1045–1052 (2014). 25218520 10.1038/nbt.2979

[b21] Li Y. *et al.* Genetic structure and diversity of cultivated soybean (*Glycine max* (L.) Merr.) landraces in China. Theor. Appl. Genet. 117, 857–871 (2008). 18587557 10.1007/s00122-008-0825-0

[b22] Lloyd J. & Meinke D. A comprehensive dataset of genes with a loss-of-function mutant phenotype in Arabidopsis. Plant Physiol. 158, 1115–1129 (2012). 22247268 10.1104/pp.111.192393 PMC3291275

[b23] Yamamoto E., Yonemaru J., Yamamoto T. & Yano M. OGRO: The Overview of functionally characterized Genes in Rice online database. Rice 5, 26 (2012). 10.1186/1939-8433-5-26 PMC5520837 27234245

[b24] Li Y. *et al.* Natural variation in *GS5* plays an important role in regulating grain size and yield in rice. Nat. Genet. 43, 1266–1269 (2011). 22019783 10.1038/ng.977

[b25] Wang E. *et al.* Control of rice grain-filling and yield by a gene with a potential signature of domestication. Nat. Genet. 40, 1370–1374 (2008). 18820698 10.1038/ng.220

[b26] Li S. *et al.* Short panicle1 encodes a putative PTR family transporter and determines rice panicle size. Plant J. 58, 592–605 (2009). 19154200 10.1111/j.1365-313X.2009.03799.x

[b27] Wang D., Tyson M. D., Jackson S. S. & Yadegari R. Partially redundant functions of two SET-domain polycomb-group proteins in controlling initiation of seed development in Arabidopsis. Proc. Natl. Acad. Sci. USA 103, 13244–13249 (2006). 16924116 10.1073/pnas.0605551103 PMC1559784

[b28] Luo M., Dennis E. S., Berger F., Peacock W. J. & Chaudhury A. *MINISEED3* (*MINI3*), a *WRKY* family gene, and *HAIKU2* (*IKU2*), a leucine-rich repeat (*LRR*) *KINASE* gene, are regulators of seed size in Arabidopsis. Proc. Natl. Acad. Sci. USA 102, 17531–17536 (2005). 16293693 10.1073/pnas.0508418102 PMC1297679

[b29] Zha X. *et al.* Over-expression of the rice *LRK1* gene improves quantitative yield components. Plant Biotechnol. J. 7, 611–620 (2009). 19619185 10.1111/j.1467-7652.2009.00428.x

[b30] Gernet J. In a history of chinese civilization. 2nd edn, (Cambridge University Press, 1996).

[b31] Guo J. *et al.* A single origin and moderate bottleneck during domestication of soybean (*Glycine max*): implications from microsatellites and nucleotide sequences. Ann. Bot. 106, 505–514 (2010). 20566681 10.1093/aob/mcq125 PMC2924825

[b32] Fleming J. E., Ellis R. H., John P., Summerfield R. J. & Roberts E. H. Developmental implications of photoperiod sensitivity in soybean (*Glycine max* [L.] Merr). Int. J. Plant. Sci. 158, 142–151 (1997).

[b33] Zhou L. *et al.* Identification of domestication-related loci associated with flowering time and seed size in soybean with the RAD-seq genotyping method. Sci. Rep. 5, 9350 (2015). 25797785 10.1038/srep09350 PMC4369735

[b34] Li D. D., Pfeiffer T. W. & Cornelius P. L. Soybean QTL for yield and yield components associated with *Glycine soja* alleles. Crop Sci. 48, 571–581 (2008).

[b35] Ding J. *et al.* Genome-wide association mapping reveals novel sources of resistance to northern corn leaf blight in maize. BMC Plant Biol. 15, 206 (2015). 26289207 10.1186/s12870-015-0589-z PMC4546088

[b36] Kertho A., Mamidi S., Bonman J. M., McClean P. E. & Acevedo M. Genome-wide association mapping for resistance to leaf and stripe rust in winter-habit hexaploid wheat landraces. PLoS One 10, e0129580 (2015). 26076040 10.1371/journal.pone.0129580 PMC4468153

[b37] Chen H. *et al.* A high-density SNP genotyping array for rice biology and molecular breeding. Mol. Plant. 7, 541–553 (2014). 24121292 10.1093/mp/sst135

[b38] Ruggieri V. *et al.* An association mapping approach to identify favourable alleles for tomato fruit quality breeding. BMC Plant Biol. 14, 337 (2014). 25465385 10.1186/s12870-014-0337-9 PMC4266912

[b39] Barrett J. C., Fry B., Maller J. & Daly M. J. Haploview: analysis and visualization of LD and haplotype maps. Bioinformatics 21, 263–265 (2005). 15297300 10.1093/bioinformatics/bth457

[b40] Cingolani P. *et al.* A program for annotating and predicting the effects of single nucleotide polymorphisms, SnpEff: SNPs in the genome of Drosophila melanogaster strain w1118; iso-2; iso-3. Fly 6, 80–92 (2012). 22728672 10.4161/fly.19695 PMC3679285

[b41] Murray M. G. & Thompson W. F. Rapid isolation of high molecular weight plant DNA. Nucleic Acids Res. 8, 4321–4325 (1980). 7433111 10.1093/nar/8.19.4321 PMC324241

[b42] Danecek P. *et al.* The variant call format and VCFtools. Bioinformatics 27, 2156–2158 (2011). 21653522 10.1093/bioinformatics/btr330 PMC3137218

[b43] Bradbury P. J. *et al.* TASSEL: software for association mapping of complex traits in diverse samples. Bioinformatics 23, 2633–2635 (2007). 17586829 10.1093/bioinformatics/btm308

[b44] Purcell S. *et al.* PLINK: a tool set for whole-genome association and population-based linkage analyses. Am. J. Hum. Genet. 81, 559–575 (2007). 17701901 10.1086/519795 PMC1950838

[b45] Alexander D. H., Novembre J. & Lange K. Fast model-based estimation of ancestry in unrelated individuals. Genome Res. 19, 1655–1664 (2009). 19648217 10.1101/gr.094052.109 PMC2752134

[b46] Lee T. H., Guo H., Wang X., Kim C. & Paterson A. H. SNPhylo: a pipeline to construct a phylogenetic tree from huge SNP data. BMC Genomics 15, 162 (2014). 24571581 10.1186/1471-2164-15-162 PMC3945939

[b47] Tamura K., Stecher G., Peterson D., Filipski A. & Kumar S. MEGA6: Molecular Evolutionary Genetics Analysis version 6.0. Mol. Biol. Evol. 30, 2725–2729 (2013). 24132122 10.1093/molbev/mst197 PMC3840312

[b48] Xu X. *et al.* Resequencing 50 accessions of cultivated and wild rice yields markers for identifying agronomically important genes. Nat. Biotechnol. 30, 105–111 (2012). 22158310 10.1038/nbt.2050

[b49] Schmutz J. *et al.* A reference genome for common bean and genome-wide analysis of dual domestications. Nat. Genet. 46, 707–713 (2014). 24908249 10.1038/ng.3008 PMC7048698

[b50] Zhang Z. *et al.* Mixed linear model approach adapted for genome-wide association studies. Nat. Genet. 42, 355–360 (2010). 20208535 10.1038/ng.546 PMC2931336

[b51] Lipka A. E. *et al.* GAPIT: genome association and prediction integrated tool. Bioinformatics 28, 2397–2399 (2012). 22796960 10.1093/bioinformatics/bts444

[b52] VanRaden P. M. Efficient methods to compute genomic predictions. J. Dairy Sci. 91, 4414–4423 (2008). 18946147 10.3168/jds.2007-0980

[b53] Yang N. *et al.* Genome wide association studies using a new nonparametric model reveal the genetic architecture of 17 agronomic traits in an enlarged maize association panel. PLoS Genet. 10, e1004573 (2014). 25211220 10.1371/journal.pgen.1004573 PMC4161304

[b54] Sun Y. N. *et al.* Multi-environment mapping and meta-analysis of 100-seed weight in soybean. Mol. Biol. Rep. 39, 9435–9443 (2012). 22740134 10.1007/s11033-012-1808-4

[b55] Orf J. H. *et al.* Genetics of soybean agronomic traits: I. Comparison of three related recombinant inbred populations. Crop Sci. 39, 1642–1651 (1999).

[b56] Han Y. P. *et al.* QTL analysis of soybean seed weight across multi-genetic backgrounds and environments. Theor. Appl. Genet. 125, 671–683 (2012). 22481120 10.1007/s00122-012-1859-x

[b57] Sebolt A. M., Shoemaker R. C. & Diers B. W. Analysis of a quantitative trait locus allele from wild soybean that increases seed protein concentration in soybean. Crop Sci. 40, 1438–1444 (2000).

[b58] Chung J. *et al.* The seed protein, oil, and yield QTL on soybean linkage group I. Crop Sci. 43, 1053–1067 (2003).

[b59] Liang Q. A., Cheng X. H., Mei M. T., Yan X. L. & Liao H. QTL analysis of root traits as related to phosphorus efficiency in soybean. Ann. Bot. 106, 223–234 (2010). 20472699 10.1093/aob/mcq097 PMC2889805

[b60] Li W., Zheng D. H., Van K. & Lee S. H. QTL mapping for major agronomic traits across two years in soybean (*Glycine max* L. Merr.). J. Crop. Sci. Biot. 11, 171–176 (2008).

[b61] Lee S. H., Park K. Y., Lee H. S., Park E. H. & Boerma H. R. Genetic mapping of QTLs conditioning soybean sprout yield and quality. Theor. Appl. Genet. 103, 702–709 (2001).

[b62] Maughan P. J., Maroof M. A. S. & Buss G. R. Molecular-marker analysis of seed weight: Genomic locations, gene action, and evidence for orthologous evolution among three legume species. Theor. Appl. Genet. 93, 574–579 (1996). 24162350 10.1007/BF00417950

[b63] Specht J. E. *et al.* Soybean response to water: A QTL analysis of drought tolerance. Crop Sci. 41, 493–509 (2001).

