# Supplementary material for: Development and application of a novel genome-wide SNP array reveals domestication history in soybean
Source: Sci Rep. 2016 Feb 9;6:20728. doi: 10.1038/srep20728 (PMC4746597; doi:10.1038/srep20728)
Supplement: Supplementary Information [file srep20728-s1.doc]

**Supplemental Information**

**Development and application of a novel genome-wide SNP array reveals domestication history in soybean**

Jiao Wang ^1^,^+^, Shanshan Chu ^1,+^, Huairen Zhang ^1,+^, Ying Zhu ^1^, Hao Cheng ^1^, Deyue Yu ^1,*^

^1^ National Center for Soybean Improvement, National Key Laboratory of Crop Genetics and Germplasm Enhancement, Nanjing Agricultural University, Nanjing, 210095, China

^*^ corresponding. [dyyu@njau.edu.cn](mailto:dyyu@njau.edu.cn)

^+^ these authors contributed equally to this work.

**
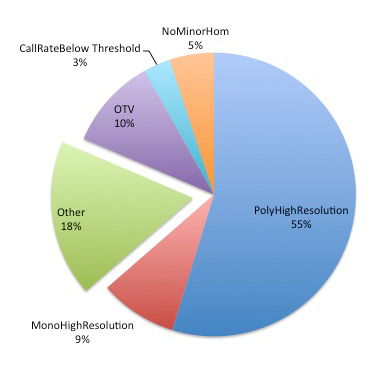
**

**Supplementary Figure S1.** Classification of SNPs in the NJAU 355K SoySNP array into six types.


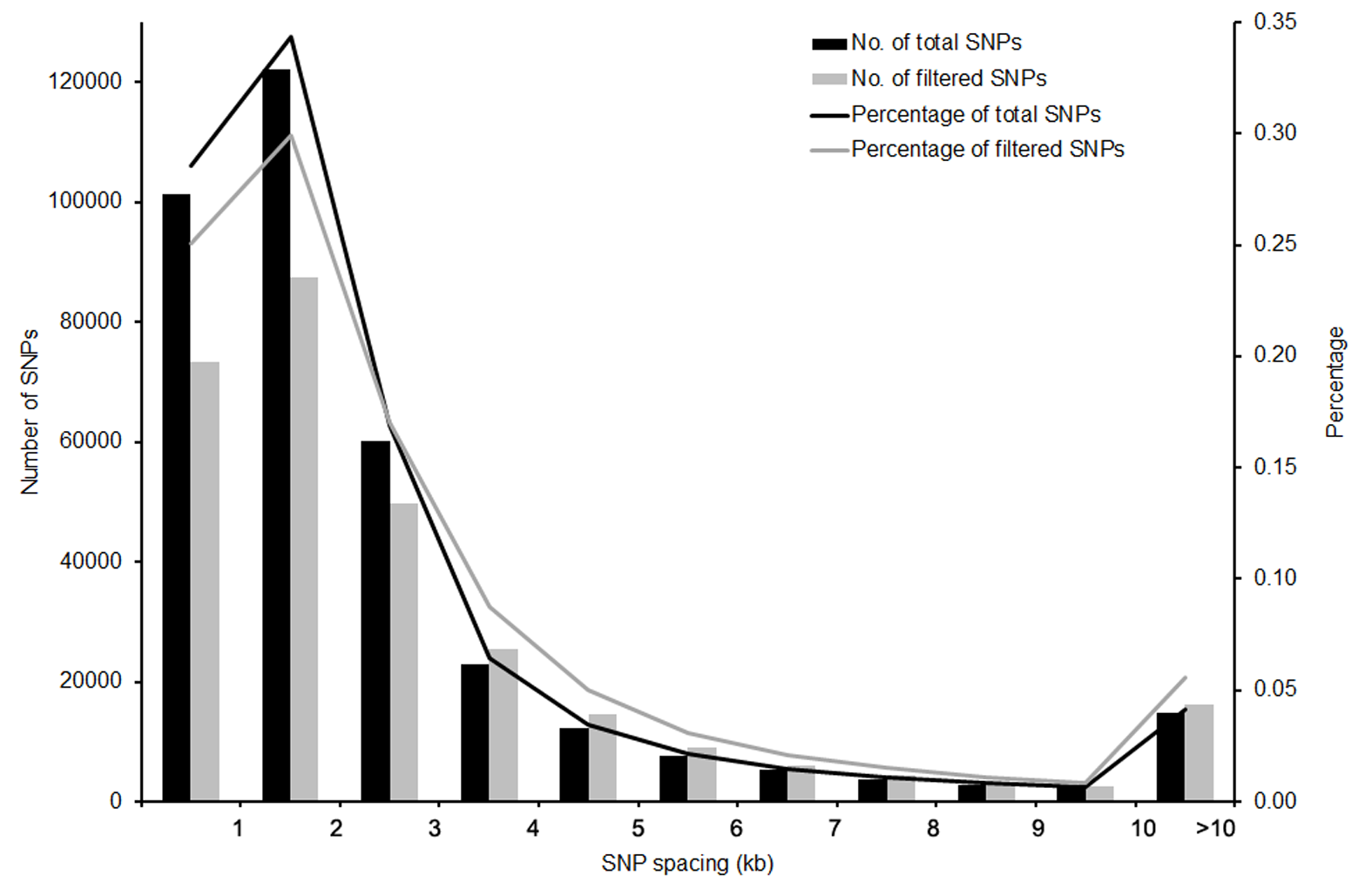


**Supplementary Figure S2.** Distribute of number and percentage of SNPs with certain spacing distance. Total SNPs indicate 355K SNPs on the array; filtered SNPs indicate the 292K filtered SNPs.


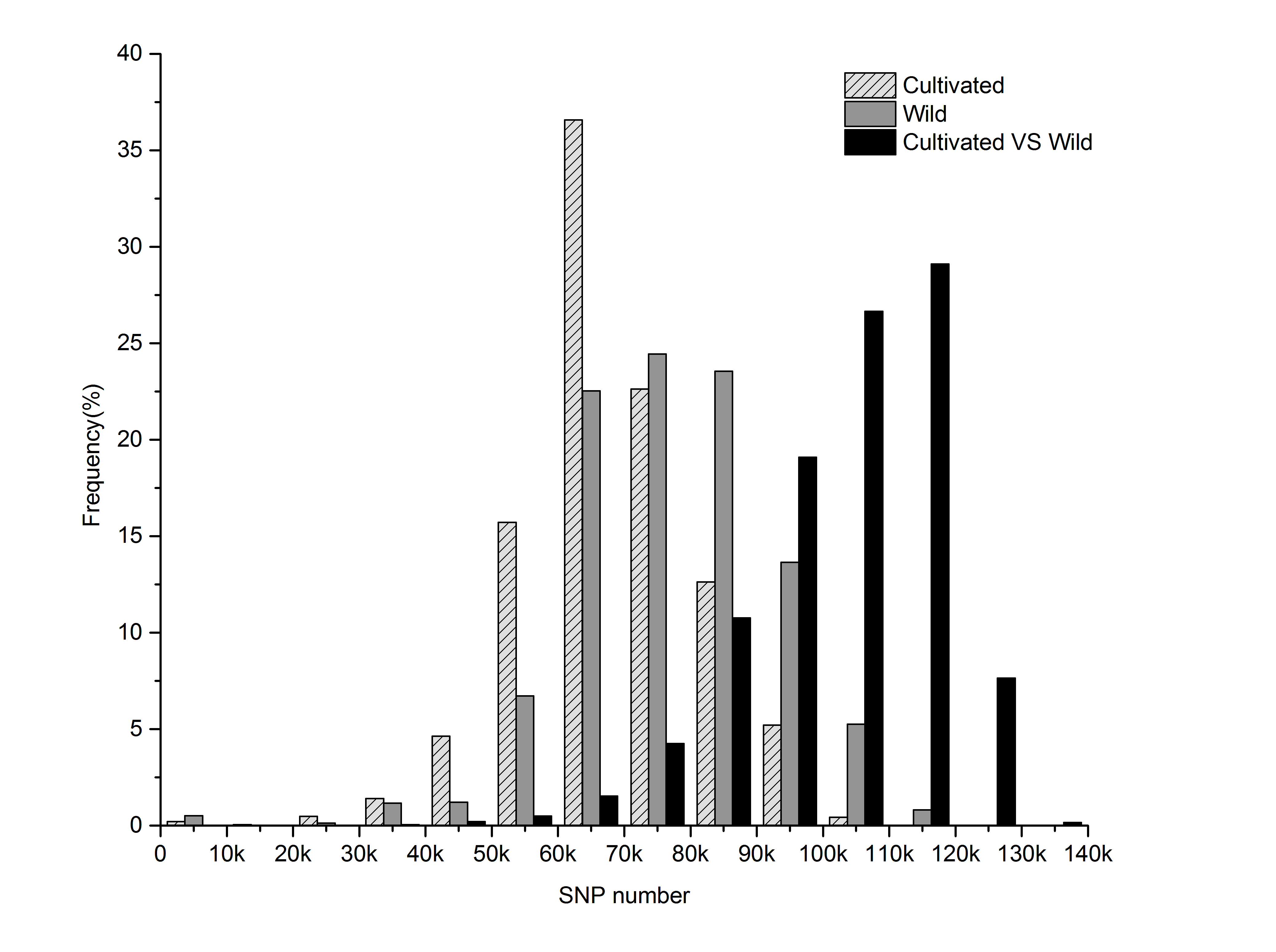


**Supplementary Figure S3.** The frequency distribution of pairwise SNP numbers. Light grey columns with slashes represent the frequency of pairwise SNP numbers of different scales in cultivated soybeans. Grey columns represent the frequency of pairwise SNP numbers of different scales in wild soybeans. Comparing cultivated and wild soybeans, we got black columns representing the frequency of pairwise SNP numbers of different scales between wild and cultivated soybeans.


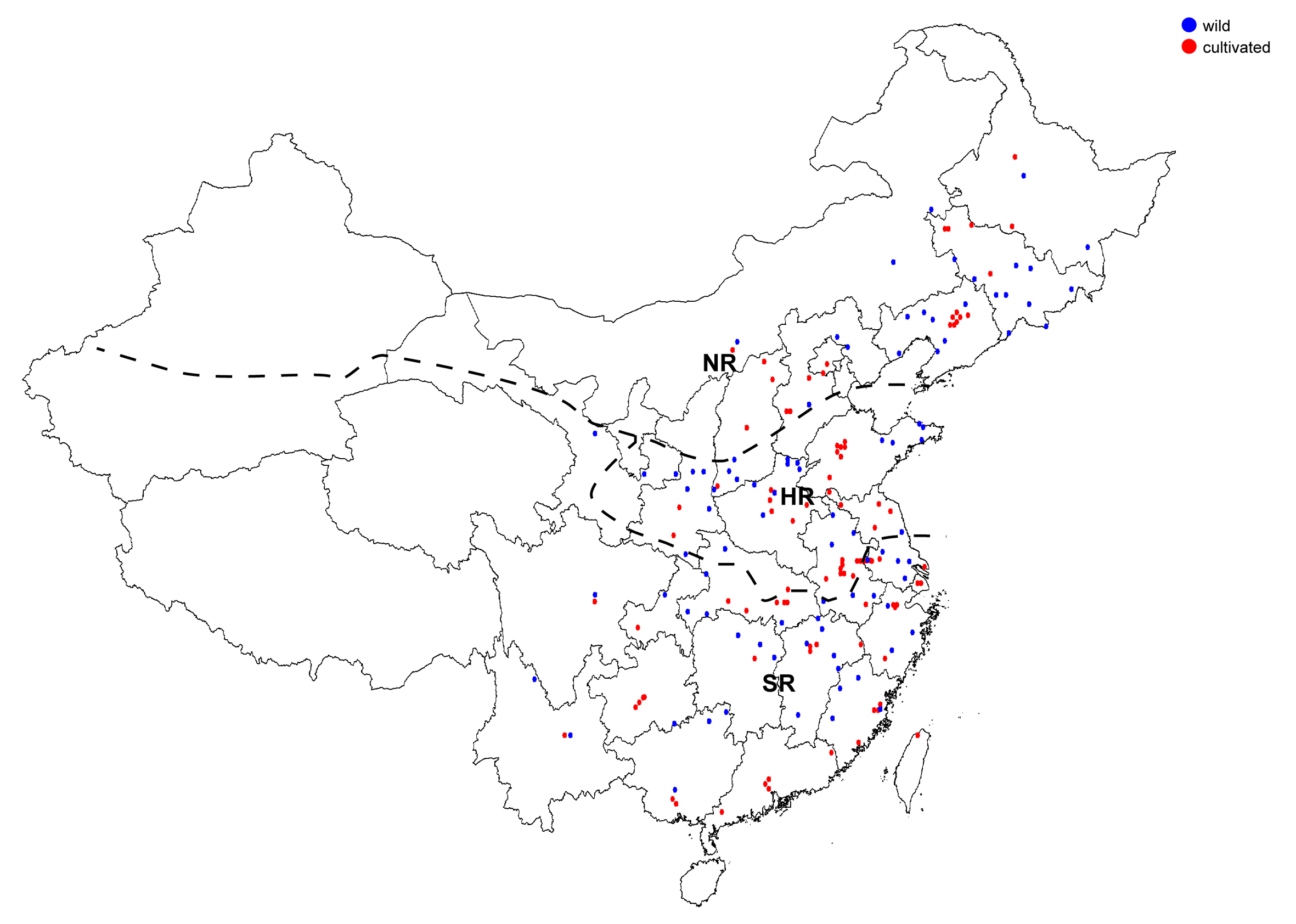


**Supplementary Figure S4.** Geographic distribution of sampled accessions. The habitat of soybean is divided into three regions, named as the northern region (NR), the Huang-Huai region (HR), and the southern region (SR). The dashed lines are the rough borders of the three ecological regions. The blue points and red points represent the wild and cultivated accessions respectively. The map was drawn using R packages maptools and ggmap (https://cran.r-project.org), and the geographic information data was downloaded from National Geomatics Center of China (http://ngcc.sbsm.gov.cn).

**
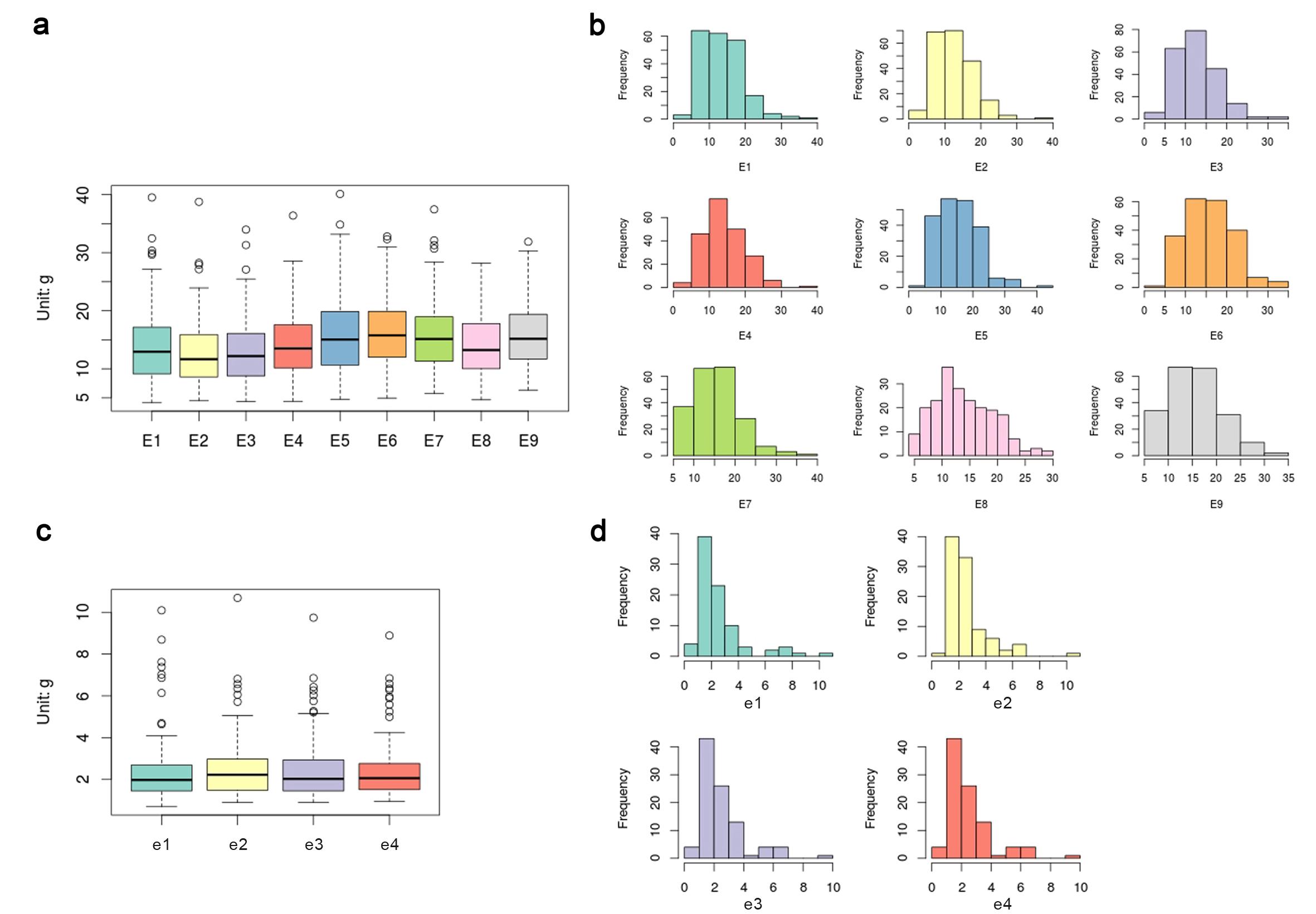
**

**Supplementary Figure S5.** Basic statistic plot and frequency distribution of variation for seed weight in different environments. (a) Basic statistic plot for cultivated soybeans. (b) Frequency distribution of variation for cultivated soybeans. (c) Basic statistic plot for wild soybeans. (d) Frequency distribution of variation for wild soybeans.

**
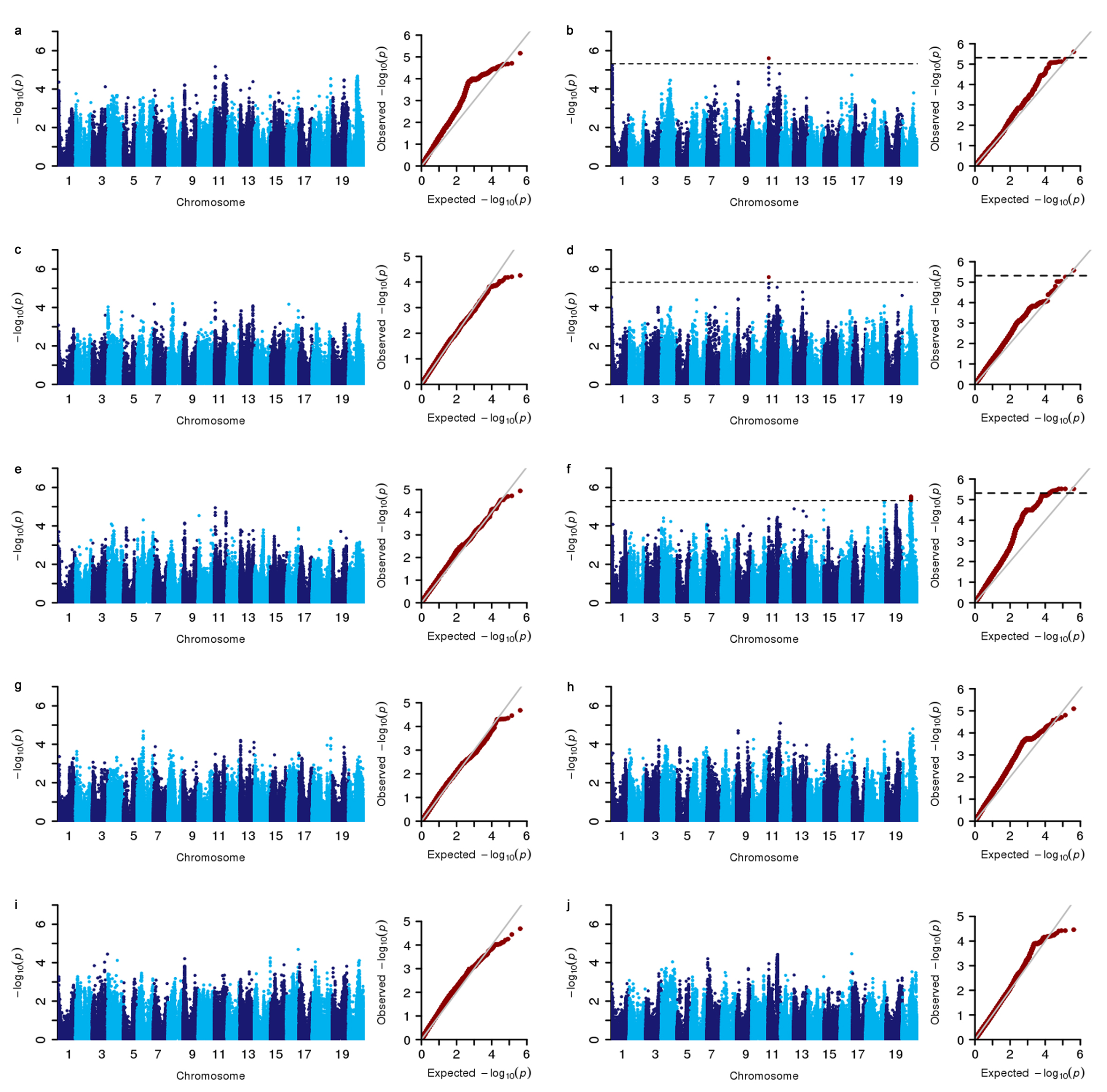
**

**Supplementary Figure S6**. Genome-wide association study of seed weight in the cultivated soybeans using the compressed MLM under multiple environments. Manhattan plots and quantile-quantile plots for seed weight in following environments (a) BLUP-C, (b) E1, (c) E2, (d) E3, (e) E4, (f) E5, (g) E6, (h) E7, (i) E8, (j) E9. The dashed horizontal line depicts significance threshold (4.82 × 10^−6^). The significant SNPs were indicated with red dots, and the candidate genes were listed in Table 2.

**
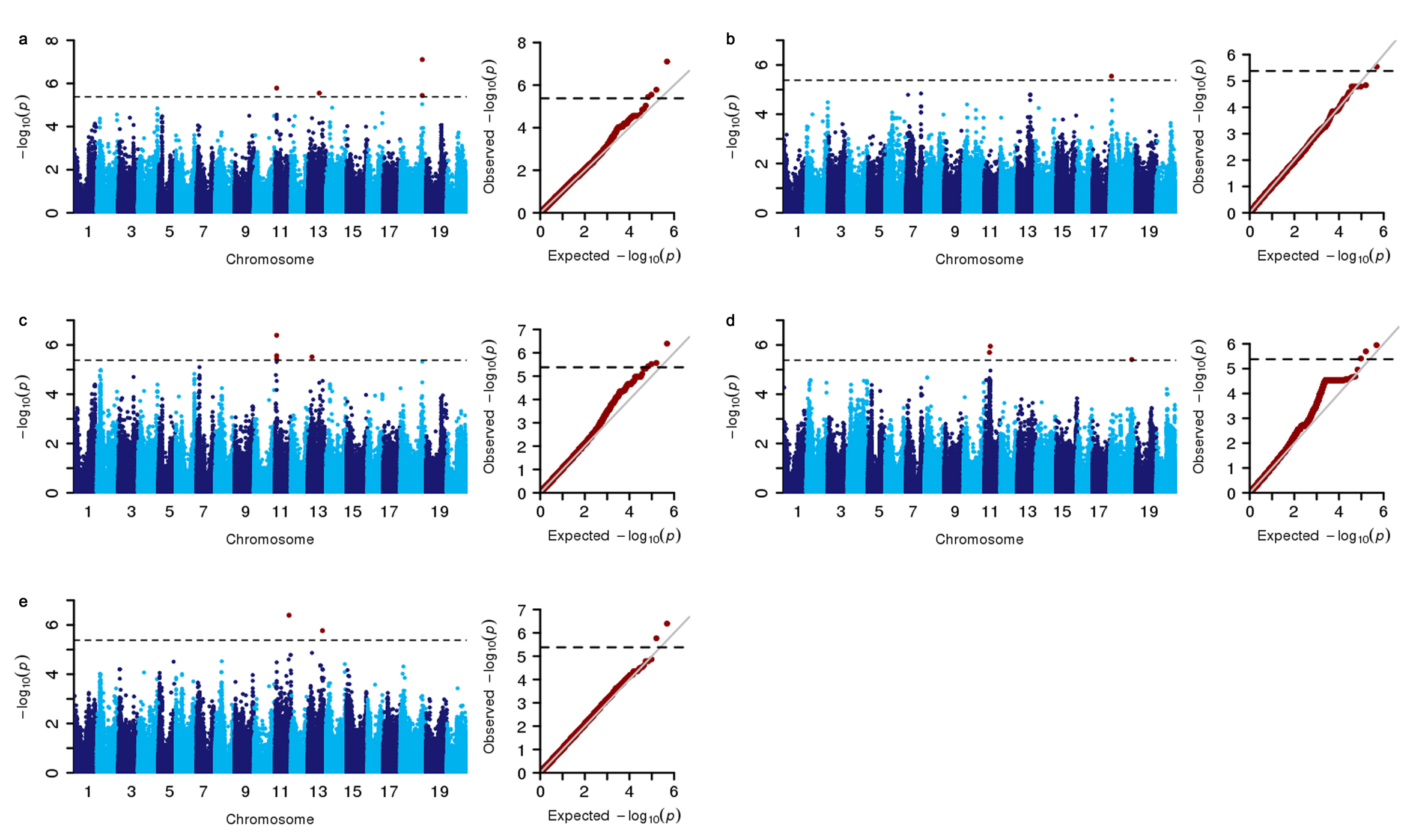
**

**Supplementary Figure S7**. Genome-wide association study of seed weight in the wild soybeans using the compressed MLM under multiple environments. Manhattan plots and quantile-quantile plots for seed weight in following environments (a) BLUP-W, (b) e1, (c) e2, (d) e3, (e) e4. The dashed horizontal line depicted significant threshold (4.17 × 10^−6^). The significant SNPs were indicated with red dots, and the candidate genes were listed in Table 2.


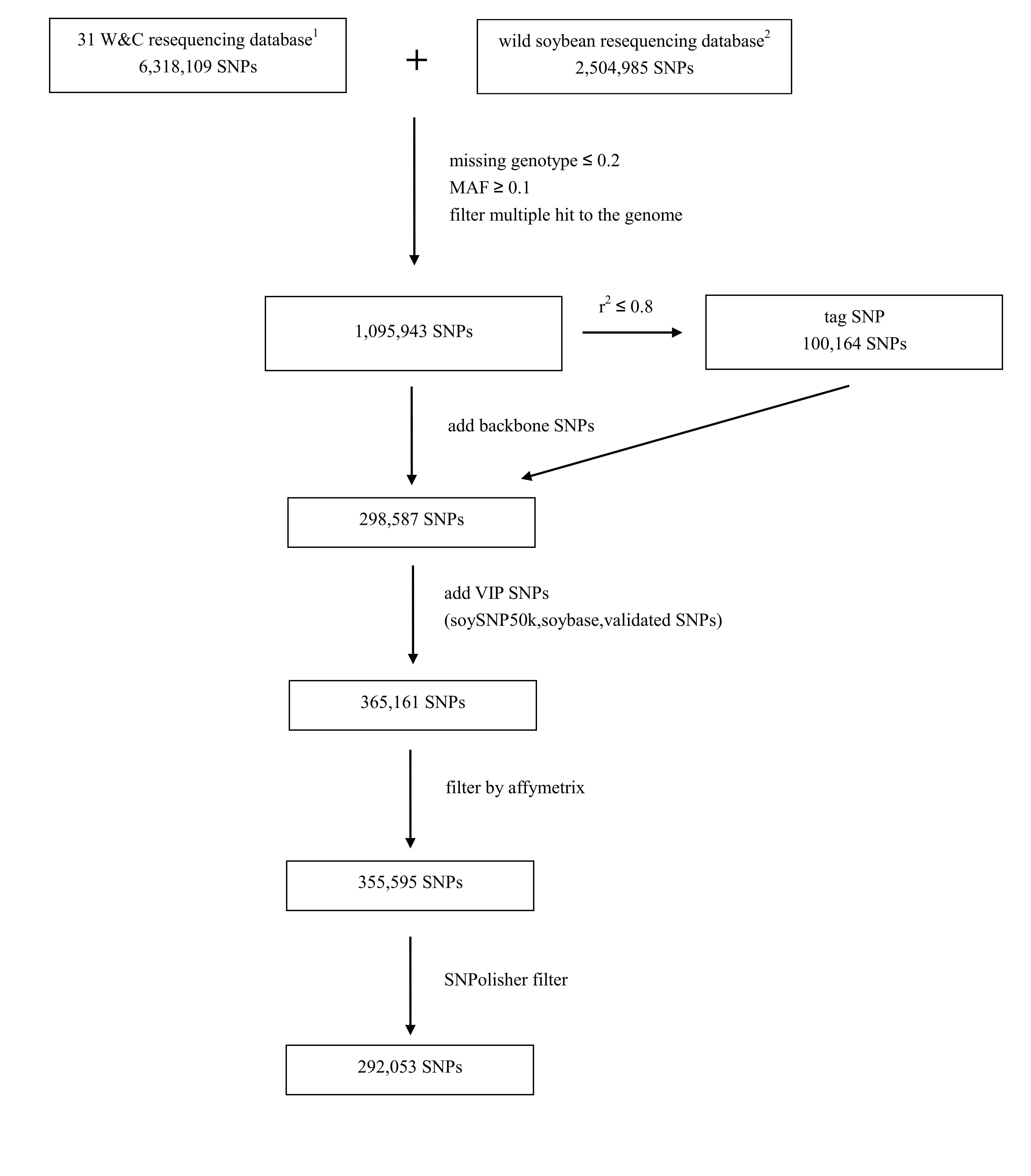


**Supplementary Figure S8.** NJAU 355K SoySNP array design pipeline.

^1^ the database derived from the paper titled “ Resequencing of 31 wild and cultivated soybean genomes identifies patterns of genetic diversity and selection ”.

^2^ the database derived from the paper titled “Whole-genome sequencing and intensive analysis of the undomesticated soybean (*Glycine soja* Sieb. and Zucc.) genome ”.

Kim MY, Lee S, Van K, Kim TH, Jeong SC, Choi IY, Kim DS, Lee YS, Park D, Ma J et al. 2010. Whole-genome sequencing and intensive analysis of the undomesticated soybean (Glycine soja Sieb. and Zucc.) genome. *Proceedings of the National Academy of Sciences of the United States of America* **107**(51): 22032-22037.

Lam HM, Xu X, Liu X, Chen W, Yang G, Wong FL, Li MW, He W, Qin N, Wang B et al. 2010. Resequencing of 31 wild and cultivated soybean genomes identifies patterns of genetic diversity and selection. *Nature genetics* **42**(12): 1053-1059.

**Supplementary Table S1. SNP distribution in soybean genome annotation.**

| Region | Count | Percent |
| --- | --- | --- |
| Intergenic | 201,874 | 69.14% |
| Exon | 25,336 | 8.68% |
| Intron | 48,993 | 16.78% |
| UTR_3_Prime | 8,874 | 3.04% |
| UTR_5_Prime | 6,885 | 2.36% |
| Total | 291,962 |  |

**Supplementary Table S2. SNP classification based on polymorphism comparison between cultivated and wild soybeans.**

| Polymorphism categories | Number | Proportion |
| --- | --- | --- |
| no_poly^a^ | 32,347 | 11.08% |
| poly_wild^b^ | 13,757 | 4.71% |
| poly_cul^c^ | 4,339 | 1.49% |
| Shared^d^ | 241,610 | 82.73% |

^a^ SNPs that show no polymorphism.

^b^ Polymorphism exist only in wild soybeans.

^c^ Polymorphism exist only in cultivated soybeans.

^d^ Polymorphism exist in both cultivated and wild soybeans.

**Supplementary Table S3.** Summary of ecological distribution of accessions of different groups.

| Ecological Regions | Number of Accessions | I | II | III | IV | V |
| --- | --- | --- | --- | --- | --- | --- |
| NR ^a^ | 47 | 0.532 | 0.191 | 0.106 | 0.085 | 0.085 |
| HR ^b^ | 86 | 0.302 | 0.279 | 0.116 | 0.198 | 0.105 |
| SR ^c^ | 193 | 0.218 | 0.026 | 0.161 | 0.389 | 0.207 |

^a^ Northern region.

^b^ Huang-Huai region.

^c^ Southern region.

**Supplementary Table S4.** Summary of 100-seed weight (g) of accessions of different evolution type from different groups.

| Evolution Type | I | | II | | III | | IV | | V | |
| --- | --- | --- | --- | --- | --- | --- | --- | --- | --- | --- |
| Wild | | 2.10 | | 4.89 | | - | | 4.74 | | 9.74 |
| Landrace | | - | | 10.59 | | 13.01 | | 14.47 | | 19.77 |
| Improved | | - | | 14.42 | | 17.59 | | 17.24 | | 23.71 |
| Total | | 2.10 | | 10.09 | | 14.50 | | 14.71 | | 20.13 |

**Supplementary Table S5.** Homologues of domestication genes in *Glycine max*.

| No. | Locus ID of  *Glycine max* | Locus ID of  *Oryza sativa* or *Arabidopsis thaliana* | Gene name of  *Oryza sativa* or *Arabidopsis thaliana* | E-value | Agronomic traits | QTL information |
| --- | --- | --- | --- | --- | --- | --- |
| 1 | *Glyma03g26261* | *AT3G54340* | *AP3* | 1.00E-40 | flowering |  |
| 2 | *Glyma03g31310* | *AT4G02700* | *SULTR3* | 0 | flowering |  |
| 3 | *Glyma04g01140* | *AT4G22140* | *EBS* | 1.00E-116 | flowering |  |
| 4 | *Glyma05g14300* | *AT1G27390* | *TOM20-2* | 9.00E-76 | flowering |  |
| 5 | *Glyma06g12380* | *AT5G20240* | *PI* | 2.00E-75 | flowering |  |
| 6 | *Glyma07g36180* | *AT1G20980* | *SPL14* | 1.00E-75 | flowering |  |
| 7 | *Glyma08g01791* | *AT4G39460* | *SAMC1* | 7.00E-45 | flowering |  |
| 8 | *Glyma08g08740* | *AT4G18240* | *AtSS4* | 0 | flowering |  |
| 9 | *Glyma08g09180* | *AT5G63980* | *FRY1* | 6.00E-171 | flowering |  |
| 10 | *Glyma08g09280* | *AT2G21070* | *FIO1* | 0 | flowering |  |
| 11 | *Glyma08g28920* | *AT2G33540* | *CPL3* | 0 | flowering |  |
| 12 | *Glyma12g03610* | *AT1G07890* | *APX1* | 4.00E-106 | flowering |  |
| 13 | *Glyma12g28690* | *LOC_Os03g60110* | *SPIN1* | 6.00E-53 | flowering |  |
| 14 | *Glyma15g00241* | *AT1G79000* | *HAC1* | 0 | flowering |  |
| 15 | *Glyma15g05970* | *AT1G22310* | *MBD8* | 9.00E-35 | flowering |  |
| 16 | *Glyma15g06491* | *AT2G22630* | *AGL17* | 3.00E-50 | flowering |  |
| 17 | *Glyma15g09260* | *LOC_Os12g38210* | *spl11* | 4.00E-80 | flowering |  |
| 18 | *Glyma19g02710* | *LOC_Os03g07360* | *OsDof12* | 1.00E-59 | flowering |  |
| 19 | *Glyma19g04326* | *AT1G69120* | *AP1* | 1.00E-27 | flowering |  |
| 20 | *Glyma01g02941* | *AT1G17110* | *UBP15* | 1.00E-121 | plant height |  |
| 21 | *Glyma02g41610* | *AT3G12380* | *ARP5* | 0 | plant height |  |
| 22 | *Glyma02g44801* | *AT5G19400* | *SMG7* | 2.00E-149 | plant height |  |
| No. | Locus ID of  *Glycine max* | Locus ID of  *Oryza sativa* or *Arabidopsis thaliana* | Gene name of  *Oryza sativa* or *Arabidopsis thaliana* | E-value | Agronomic traits | QTL information |
| 23 | *Glyma03g26285* | *AT5G17690* | *TFL2* | 2.00E-24 | plant height |  |
| 24 | *Glyma03g36350* | *AT5G48910* | *LPA66* | 1.00E-165 | plant height |  |
| 25 | *Glyma05g07050* | *AT1G66980* | *SNC4* | 2.00E-60 | plant height |  |
| 26 | *Glyma05g12833* | *AT1G49430* | *LACS2* | 2.00E-41 | plant height |  |
| 27 | *Glyma05g13910* | *LOC_Os11g05110* | *OsPK1* | 1.00E-70 | plant height |  |
| 28 | *Glyma05g14140* | *LOC_Os04g51350* | *MPR25* | 3.00E-89 | plant height |  |
| 29 | *Glyma05g14170* | *AT2G26990* | *FUS12* | 0 | plant height |  |
| 30 | *Glyma05g14700* | *LOC_Os07g49400* | *OsApx2* | 2.00E-22 | plant height |  |
| 31 | *Glyma06g02930* | *LOC_Os04g48760* | *xiao* | 0 | plant height |  |
| 32 | *Glyma06g02940* | *LOC_Os01g33040* | *dbs1* | 2.00E-159 | plant height |  |
| 33 | *Glyma06g12350* | *AT3G21070* | *NADK1* | 1.00E-29 | plant height |  |
| 34 | *Glyma06g14750* | *LOC_Os04g57720* | *OsRR6* | 4.00E-37 | plant height |  |
| 35 | *Glyma07g34880* | *AT5G49830* | *EXO84B* | 2.00E-102 | plant height |  |
| 36 | *Glyma07g35780* | *AT5G66350* | *SHI* | 3.00E-42 | plant height |  |
| 37 | *Glyma07g36655* | *AT5G49510* | *PFD3* | 2.00E-36 | plant height |  |
| 38 | *Glyma08g00680* | *AT1G28380* | *NSL1* | 0 | plant height |  |
| 39 | *Glyma08g06020* | *LOC_Os01g52050* | *d61* | 3.00E-67 | plant height |  |
| 40 | *Glyma08g09040* | *LOC_Os01g55240* | *GA2ox3* | 3.00E-91 | plant height |  |
| 41 | *Glyma08g09050* | *AT1G80350* | *FRA2* | 6.00E-93 | plant height |  |
| 42 | *Glyma08g28391* | *AT1G30100* | *NCED5* | 1.00E-42 | plant height |  |
| 43 | *Glyma08g47941* | *AT1G67140* | *SWEETIE* | 4.00E-35 | plant height |  |
| 44 | *Glyma10g33380* | *LOC_Os01g45860* | *SLRL1* | 2.00E-127 | plant height |  |
| 45 | *Glyma10g34020* | *LOC_Os11g01330* | *D53* | 3.00E-28 | plant height |  |
| No. | Locus ID of  *Glycine max* | Locus ID of  *Oryza sativa* or *Arabidopsis thaliana* | Gene name of  *Oryza sativa* or *Arabidopsis thaliana* | E-value | Agronomic traits | QTL information |
| 46 | *Glyma11g35990* | *AT4G39640* | *GGT1* | 0 | plant height |  |
| 47 | *Glyma12g14296* | *AT1G06230* | *GTE4* | 5.00E-137 | plant height |  |
| 48 | *Glyma12g33280* | *AT5G17400* | *ER-ANT1* | 2.00E-120 | plant height |  |
| 49 | *Glyma13g00660* | *AT2G19080* | *METAXIN* | 4.00E-83 | plant height |  |
| 50 | *Glyma13g20460* | *AT2G22410* | *SLO1* | 1.00E-137 | plant height |  |
| 51 | *Glyma13g31490* | *AT3G26940* | *CDG1* | 4.00E-84 | plant height |  |
| 52 | *Glyma13g41261* | *LOC_Os03g49990* | *Slr1-d* | 2.00E-39 | plant height |  |
| 53 | *Glyma19g35460* | *AT2G20190* | *CLASP* | 0 | plant height |  |
| 54 | *Glyma04g01350* | *LOC_Os01g44220* | *osagpl2* | 2.00E-137 | seed composition |  |
| 55 | *Glyma05g05860* | *LOC_Os12g43550* | *gpa1* | 4.00E-37 | seed composition |  |
| 56 | *Glyma07g39040* | *LOC_Os08g09230* | *ss3a* | 0 | seed composition |  |
| 57 | *Glyma11g09420* | *AT5G67360* | *ARA12* | 3.00E-174 | seed composition |  |
| 58 | *Glyma15g05720* | *AT5G49360* | *BXL1* | 0 | seed composition |  |
| 59 | *Glyma15g06400* | *LOC_Os08g32870* | *Badh2* | 3.00E-54 | seed composition |  |
| 60 | *Glyma17g01900* | *LOC_Os08g25734* | *osagps2* | 1.00E-148 | seed composition |  |
| 61 | *Glyma17g12530* | *AT5G17770* | *AtCBR* | 5.00E-59 | seed composition |  |
| 62 | *Glyma01g40590* | *AT1G75820* | *CLV1* | 0 | seed weight |  |
| 63 | *Glyma01g41990* | *LOC_Os04g33740* | *GIF1* | 7.00E-130 | seed weight | sw^a^ 29-3,sw 30-1 |
| 64 | *Glyma06g02738* | *AT2G36010_E2Fa* | *seed* | 4.00E-54 | seed weight |  |
| 65 | *Glyma06g03950* | *LOC_Os11g12740* | *sp1* | 6.00E-115 | seed weight |  |
| 66 | *Glyma06g12270* | *AT5G49630* | *AAP6* | 0 | seed weight |  |
| 67 | *Glyma06g12800* | *LOC_Os05g06660* | *GS5* | 2.00E-44 | seed weight | swi^b^ 1-4,sl^c^ 3-1,sw 33-1,swi 3-1 |
| 68 | *Glyma07g35690* | *LOC_Os01g15900* | *Rdd1* | 1.00E-72 | seed weight | sw 12-1 |
| No. | Locus ID of  *Glycine max* | Locus ID of  *Oryza sativa* or *Arabidopsis thaliana* | Gene name of  *Oryza sativa* or *Arabidopsis thaliana* | E-value | Agronomic traits | QTL information |
| 69 | *Glyma07g36630* | *LOC_Os09g03610* | *rFCA* | 5.00E-153 | seed weight | sw 12-1 |
| 70 | *Glyma08g01390* | *AT5G50600_HSD1* | *seed* | 4.00E-105 | seed weight |  |
| 71 | *Glyma12g33450* | *At3g19700* | *HAIKU2/IKU2* | 7E-166 | seed weight | sw 13-8, sw 34-4 |
| 72 | *Glyma13g35491* | *AT1G52920* | *GCR2* | 2.00E-111 | seed weight |  |
| 73 | *Glyma13g35520* | *LOC_Os07g42410* | *srs1* | 2.00E-36 | seed weight | sy^d^ 5-3 |
| 74 | *Glyma13g36310* | *AT3G20740* | *FIE/FIS3* | 3.00E-167 | seed weight |  |
| 75 | *Glyma14g36520* | *LOC_Os02g36570* | *OsABC1-2* | 4.00E-39 | seed weight |  |
| 76 | *Glyma19g02810* | *AT1G26770* | *EXP10* | 4.00E-115 | seed weight |  |
| 77 | *Glyma19g38970* | *AT3G13220* | *ABCG26* | 0 | seed weight |  |
| 78 | *Glyma01g21120* | *LOC_Os07g48820* | *rtGA2.1* | 8.00E-118 | stress response |  |
| 79 | *Glyma01g21707* | *AT1G25540* | *PFT1* | 6.00E-170 | stress response |  |
| 80 | *Glyma01g34370* | *AT1G48050* | *KU80* | 7.00E-54 | stress response |  |
| 81 | *Glyma01g40400* | *LOC_Os04g48850* | *OsACS2* | 3.00E-138 | stress response |  |
| 82 | *Glyma01g40590* | *LOC_Os03g12730* | *OsBRR1* | 0 | stress response |  |
| 83 | *Glyma01g41670* | *LOC_Os09g36930* | *OsPIP2;7* | 5.00E-33 | stress response |  |
| 84 | *Glyma01g41930* | *AT1G12110* | *CHL1* | 0 | stress response |  |
| 85 | *Glyma01g41970* | *AT1G27450* | *APT1* | 2.00E-69 | stress response |  |
| 86 | *Glyma01g42150* | *LOC_Os10g08580* | *qUVR-10* | 8.00E-121 | stress response |  |
| 87 | *Glyma02g01730* | *AT3G44110* | *J3* | 1.00E-41 | stress response |  |
| 88 | *Glyma03g34970* | *LOC_Os01g73770* | *OsDREB1F* | 1.00E-36 | stress response |  |
| 89 | *Glyma03g35040* | *AT1G15520* | *ABCG40* | 0 | stress response |  |
| 90 | *Glyma04g01380* | *LOC_Os01g01660* | *OsIRL* | 7.00E-97 | stress response |  |
| 91 | *Glyma04g04355* | *AT1G46768* | *RAP2.1* | 7.00E-49 | stress response |  |
| No. | Locus ID of  *Glycine max* | Locus ID of  *Oryza sativa* or *Arabidopsis thaliana* | Gene name of  *Oryza sativa* or *Arabidopsis thaliana* | E-value | Agronomic traits | QTL information |
| 92 | *Glyma05g04950* | *LOC_Os10g41200* | *MYBS3* | 3.00E-67 | stress response |  |
| 93 | *Glyma05g05030* | *AT5G47650* | *NUDX2* | 1.00E-85 | stress response |  |
| 94 | *Glyma05g05130* | *AT1G06160* | *ORA59* | 7.00E-21 | stress response |  |
| 95 | *Glyma05g05180* | *LOC_Os01g58420* | *AP37* | 3.00E-21 | stress response |  |
| 96 | *Glyma05g06420* | *LOC_Os08g03290* | *OsGAPC3* | 3.00E-146 | stress response |  |
| 97 | *Glyma05g10202* | *AT4G04720* | *CPK21* | 8.00E-120 | stress response |  |
| 98 | *Glyma05g13580* | *AT4G23650* | *CPK3* | 7.00E-36 | stress response |  |
| 99 | *Glyma05g13890* | *LOC_Os02g52780* | *OsbZIP23* | 3.00E-40 | stress response |  |
| 100 | *Glyma05g13900* | *AT3G56800* | *CaM3* | 2.00E-106 | stress response |  |
| 101 | *Glyma05g14240* | *AT1G74920* | *ALDH10A8* | 2.00E-25 | stress response |  |
| 102 | *Glyma05g14330* | *AT1G53850* | *PAE1* | 3.00E-41 | stress response |  |
| 103 | *Glyma05g14420* | *AT1G37130* | *NIA2* | 2.00E-27 | stress response |  |
| 104 | *Glyma05g14850* | *LOC_Os01g04380* | *OsHsp17.0* | 4.00E-44 | stress response |  |
| 105 | *Glyma05g17340* | *LOC_Os04g38480* | *OsSERK1* | 3.00E-84 | stress response |  |
| 106 | *Glyma05g18410* | *AT5G67160* | *EPS1* | 8.00E-93 | stress response |  |
| 107 | *Glyma06g02580* | *LOC_Os02g02840* | *OsRacB* | 2.00E-103 | stress response |  |
| 108 | *Glyma06g03050* | *AT4G39090* | *RD19* | 0 | stress response |  |
| 109 | *Glyma06g03960* | *AT1G64030* | *SRP3* | 1.00E-111 | stress response |  |
| 110 | *Glyma06g09830* | *LOC_Os01g08330* | *OsCDR1* | 1.00E-21 | stress response |  |
| 111 | *Glyma06g12340* | *LOC_Os05g05680* | *OsACO5* | 1.00E-110 | stress response |  |
| 112 | *Glyma06g12780* | *LOC_Os11g10480* | *rad* | 8.00E-146 | stress response |  |
| 113 | *Glyma07g34900* | *AT4G32150* | *AtVAMP711* | 3.00E-38 | stress response |  |
| 114 | *Glyma07g35581* | *LOC_Os01g62410* | *OsMYB3R-2* | 4.00E-30 | stress response |  |
| No. | Locus ID of  *Glycine max* | Locus ID of  *Oryza sativa* or *Arabidopsis thaliana* | Gene name of  *Oryza sativa* or *Arabidopsis thaliana* | E-value | Agronomic traits | QTL information |
| 115 | *Glyma07g35700* | *LOC_Os07g25710* | *OsPHR2* | 4.00E-29 | stress response |  |
| 116 | *Glyma07g36166* | *AT3G53480* | *PDR9* | 0 | stress response |  |
| 117 | *Glyma07g36370* | *AT4G11660* | *HsfB2b* | 3.00E-50 | stress response |  |
| 118 | *Glyma07g36430* | *LOC_Os03g20090* | *OsMYB2* | 2.00E-75 | stress response |  |
| 119 | *Glyma07g36640* | *LOC_Os01g43650* | *OsWRKY11* | 3.00E-46 | stress response |  |
| 120 | *Glyma07g36740* | *LOC_Os03g60650* | *Xb15* | 3.00E-33 | stress response |  |
| 121 | *Glyma08g00880* | *LOC_Os01g53294* | *OsrbohA* | 0 | stress response |  |
| 122 | *Glyma08g09460* | *LOC_Os03g55240* | *bel* | 5.00E-110 | stress response |  |
| 123 | *Glyma10g44212* | *LOC_Os08g42580* | *OsCERK1* | 2.00E-53 | stress response |  |
| 124 | *Glyma11g04220* | *LOC_Os01g64970* | *SAPK4* | 3.00E-41 | stress response |  |
| 125 | *Glyma11g13780* | *AT2G44490* | *PEN2* | 3.00E-171 | stress response |  |
| 126 | *Glyma11g34130* | *LOC_Os03g16570* | *OsSDIR1* | 1.00E-108 | stress response |  |
| 127 | *Glyma11g34940* | *LOC_Os01g63420* | *OsCOI1* | 0 | stress response |  |
| 128 | *Glyma11g36010* | *LOC_Os04g54474* | *ostgap1* | 4.00E-110 | stress response |  |
| 129 | *Glyma12g03620* | *AT2G20990* | *SYTA* | 0 | stress response |  |
| 130 | *Glyma12g28730* | *LOC_Os01g10840* | *OsGSK1* | 4.00E-162 | stress response |  |
| 131 | *Glyma12g31940* | *AT3G07630* | *ADT2* | 2.00E-130 | stress response |  |
| 132 | *Glyma13g00840* | *LOC_Os03g01120* | *Osa8* | 0 | stress response |  |
| 133 | *Glyma13g16650* | *AT4G26070* | *MEK1* | 2.00E-165 | stress response |  |
| 134 | *Glyma13g21480* | *LOC_Os03g06410* | *OsACDR1* | 1.00E-100 | stress response |  |
| 135 | *Glyma13g21490* | *AT2G26150* | *HsfA2* | 2.00E-57 | stress response |  |
| 136 | *Glyma13g21560* | *LOC_Os02g43970* | *ARAG1* | 2.00E-31 | stress response |  |
| 137 | *Glyma13g35915* | *AT1G11350* | *CBRLK1* | 0 | stress response |  |
| No. | Locus ID of  *Glycine max* | Locus ID of  *Oryza sativa* or *Arabidopsis thaliana* | Gene name of  *Oryza sativa* or *Arabidopsis thaliana* | E-value | Agronomic traits | QTL information |
| 138 | *Glyma13g35987* | *LOC_Os06g29810* | *Pi-d2* | 1.00E-79 | stress response |  |
| 139 | *Glyma13g42720* | *AT1G55180* | *PLDE* | 8.00E-70 | stress response |  |
| 140 | *Glyma14g36430* | *AT3G56400* | *WRKY70* | 1.00E-34 | stress response |  |
| 141 | *Glyma14g36446* | *LOC_Os05g25770* | *OsWRKY45* | 3.00E-27 | stress response |  |
| 142 | *Glyma14g36462* | *AT4G14850* | *LOI1* | 6.00E-62 | stress response |  |
| 143 | *Glyma15g00340* | *AT2G41560* | *ACA4* | 0 | stress response |  |
| 144 | *Glyma15g05580* | *LOC_Os04g09920* | *CYP99A3* | 3.00E-106 | stress response |  |
| 145 | *Glyma15g05700* | *AT1G22400* | *UGT85A1* | 0 | stress response |  |
| 146 | *Glyma15g05910* | *AT2G20630* | *PIA1* | 6.00E-75 | stress response |  |
| 147 | *Glyma15g07510* | *AT1G61210* | *DWA3* | 0 | stress response |  |
| 148 | *Glyma17g09390* | *AT3G27660* | *OLEO4* | 3.00E-22 | stress response |  |
| 149 | *Glyma17g09530* | *AT5G46330* | *FLS2* | 1.00E-110 | stress response |  |
| 150 | *Glyma17g09680* | *LOC_Os03g12840* | *dsm3* | 1.00E-61 | stress response |  |
| 151 | *Glyma17g09770* | *AT1G73660* | *Resistant* | 8.00E-54 | stress response |  |
| 152 | *Glyma19g02801* | *AT2G39660* | *BIK1* | 7.00E-29 | stress response |  |
| 153 | *Glyma19g04390* | *LOC_Os10g38060* | *OsPLDbeta1* | 8.00E-42 | stress response |  |
| 154 | *Glyma19g04870* | *AT5G54590* | *CRLK1* | 4.00E-72 | stress response |  |
| 155 | *Glyma19g32940* | *AT3G12120* | *FAD2* | 0 | stress response |  |
| 156 | *Glyma19g35320* | *AT2G20310* | *RIN13* | 2.00E-30 | stress response |  |
| 157 | *Glyma19g35740* | *LOC_Os01g62190* | *ZFP179* | 2.00E-22 | stress response |  |
| 158 | *Glyma19g35890* | *AT3G12360* | *ITN1* | 0 | stress response |  |
| 159 | *Glyma19g36270* | *AT3G09710* | *IQD1* | 8.00E-83 | stress response |  |
| 160 | *Glyma19g36370* | *AT1G11310* | *MLO2* | 8.00E-124 | stress response |  |
| No. | Locus ID of  *Glycine max* | Locus ID of  *Oryza sativa* or *Arabidopsis thaliana* | Gene name of  *Oryza sativa* or *Arabidopsis thaliana* | E-value | Agronomic traits | QTL information |
| 161 | *Glyma19g36410* | *LOC_Os05g48020* | *OsSYP71* | 7.00E-102 | stress response |  |
| 162 | *Glyma19g36421* | *LOC_Os01g60020* | *OsNAC4* | 2.00E-64 | stress response |  |
| 163 | *Glyma19g40820* | *AT5G13160* | *PBS1* | 5.00E-85 | stress response |  |

^a^ sw means “seed weight”.

^b^ swi means “seed width”.

^c^ sl means “seed length”.

^d^ sy means “seed yield”.

**Supplementary Table S6.** Description of basic statistics and heritability of seed weight in cultivated soybeans

| Trait | Environment | Mean | Sd^a^ | Median | Min | Max | Range | Skew | Kurtosis | Se^b^ | Heritability |
| --- | --- | --- | --- | --- | --- | --- | --- | --- | --- | --- | --- |
| SW | E1 | 13.64 | 5.75 | 12.94 | 4.15 | 39.51 | 35.36 | 1.01 | 1.71 | 0.4 | 83.8 |
|  | E2 | 12.51 | 5.27 | 11.65 | 4.5 | 38.76 | 34.26 | 1.03 | 2.22 | 0.36 | 94.6 |
|  | E3 | 12.79 | 5.13 | 12.19 | 4.37 | 33.97 | 29.61 | 0.86 | 1.1 | 0.35 | 84 |
|  | E4 | 14.05 | 5.38 | 13.5 | 4.41 | 36.4 | 31.99 | 0.66 | 0.59 | 0.37 | 78.5 |
|  | E5 | 15.69 | 6.36 | 15.05 | 4.76 | 40.11 | 35.35 | 0.63 | 0.52 | 0.44 | 78 |
|  | E6 | 16.01 | 5.63 | 15.76 | 4.96 | 32.8 | 27.84 | 0.36 | -0.05 | 0.39 | 82.4 |
|  | E7 | 15.42 | 5.61 | 15.13 | 5.72 | 37.46 | 31.74 | 0.71 | 0.67 | 0.39 | 79.1 |
|  | E8 | 13.88 | 5.25 | 13.24 | 4.67 | 28.18 | 23.52 | 0.43 | -0.46 | 0.36 | 63.9 |
|  | E9 | 15.75 | 5.4 | 15.17 | 6.34 | 31.89 | 25.55 | 0.51 | -0.22 | 0.37 | 85.9 |

^a^ “Sd” means standard deviation.

^b^ “Se” means standard error.

**Supplementary Table S7.** Description of basic statistics and heritability of seed weight in wild soybeans

| Trait | Environment | Mean | Sd^a^ | Median | Min | Max | Range | Skew | Kurtosis | Se^b^ | Heritability |
| --- | --- | --- | --- | --- | --- | --- | --- | --- | --- | --- | --- |
| SW | e1 | 2.56 | 1.8 | 1.98 | 0.7 | 10.09 | 9.4 | 2.17 | 4.7 | 0.19 | 93.9 |
|  | e2 | 2.55 | 1.55 | 2.22 | 0.9 | 10.69 | 9.79 | 2.29 | 7.18 | 0.16 | 90.9 |
|  | e3 | 2.44 | 1.53 | 2.03 | 0.9 | 9.74 | 8.85 | 2.05 | 5.05 | 0.16 | 71.8 |
|  | e4 | 2.48 | 1.49 | 2.06 | 0.95 | 8.89 | 7.94 | 2 | 3.93 | 0.15 | 99 |

^a^ “Sd” means standard deviation.

^b^ “Se” means standard error.

**Supplementary Table S8.** 367 soybean accessions included in this study.

| No. | Accession | Origin^a^ | Evolution Type | Soybean ecological regions in China |
| --- | --- | --- | --- | --- |
| 1 | NJAU_C001 | Nanjing | Improved | III |
| 2 | NJAU_C002 | Nanjing | Improved | III |
| 3 | NJAU_C003 | Yichang | landrace | III |
| 4 | NJAU_C004 | Shanxi | landrace | -^b^ |
| 5 | NJAU_C005 | Jiangxi | landrace | III |
| 6 | NJAU_C006 | Jurong | landrace | III |
| 7 | NJAU_C007 | Fengxian | landrace | II |
| 8 | NJAU_C008 | He'nan | landrace | II |
| 9 | NJAU_C009 | He'nan | landrace | II |
| 10 | NJAU_C010 | Sichuan | landrace | III |
| 11 | NJAU_C012 | Hubei | landrace | III |
| 12 | NJAU_C013 | Guangdong | landrace | III |
| 13 | NJAU_C014 | Zhenjiang | landrace | III |
| 14 | NJAU_C015 | Jiangsu | landrace | III |
| 15 | NJAU_C016 | Zhejiang | landrace | III |
| 16 | NJAU_C017 | He'nan | landrace | II |
| 17 | NJAU_C018 | Guizhou | landrace | III |
| 18 | NJAU_C019 | Zhejiang | landrace | III |
| 19 | NJAU_C020 | Zhejiang | landrace | III |
| 20 | NJAU_C021 | Shanxi | landrace | - |
| 21 | NJAU_C022 | Fujian | landrace | III |
| 22 | NJAU_C023 | Jiangsu | landrace | III |
| 23 | NJAU_C024 | Shanghai | landrace | III |
| 24 | NJAU_C025 | Jiangxi | landrace | III |
| 25 | NJAU_C026 | Yushan | landrace | III |
| 26 | NJAU_C027 | Shiquan | landrace | III |
| 27 | NJAU_C028 | Fujian | landrace | III |
| 28 | NJAU_C029 | Daan | landrace | I |
| 29 | NJAU_C030 | Jiangsu | landrace | III |
| 30 | NJAU_C031 | Jiangxi | landrace | III |
| 31 | NJAU_C032 | Guangxi | landrace | III |
| 32 | NJAU_C033 | Hebei | landrace | II |
| 33 | NJAU_C034 | Hubei | landrace | III |
| 34 | NJAU_C035 | Neimenggu | landrace | I |
| 35 | NJAU_C036 | Jiangxi | landrace | III |
| 36 | NJAU_C037 | Guangxi | landrace | III |
| 37 | NJAU_C038 | Sichuan | landrace | III |
| 38 | NJAU_C039 | Jiangsu | landrace | III |
| 39 | NJAU_C040 | Sichuan | landrace | III |
| 40 | NJAU_C041 | Hubei | landrace | III |
| No. | Accession | Origin^a^ | Evolution Type | Soybean ecological regions in China |
| 41 | NJAU_C043 | Hunan | landrace | III |
| 42 | NJAU_C044 | Jiangxi | landrace | III |
| 43 | NJAU_C045 | Jiangsu | Improved | III |
| 44 | NJAU_C046 | Huaiyin | landrace | III |
| 45 | NJAU_C047 | Yunnan | landrace | III |
| 46 | NJAU_C048 | Shanghai | landrace | III |
| 47 | NJAU_C049 | Yunnan | landrace | III |
| 48 | NJAU_C050 | Liaoning | landrace | I |
| 49 | NJAU_C051 | Hubei | landrace | III |
| 50 | NJAU_C052 | Jiangxi | landrace | III |
| 51 | NJAU_C053 | Jiangxi | landrace | III |
| 52 | NJAU_C054 | Guangdong | landrace | III |
| 53 | NJAU_C055 | Guangxi | landrace | III |
| 54 | NJAU_C056 | - | landrace | - |
| 55 | NJAU_C057 | - | landrace | - |
| 56 | NJAU_C058 | Jiangsu | landrace | III |
| 57 | NJAU_C059 | Jilin | landrace | I |
| 58 | NJAU_C060 | Guizhou | landrace | III |
| 59 | NJAU_C061 | Yuncheng | landrace | II |
| 60 | NJAU_C062 | Heilongjiang | landrace | I |
| 61 | NJAU_C063 | Lingqiu | landrace | I |
| 62 | NJAU_C064 | Zhenba | landrace | III |
| 63 | NJAU_C065 | Hebei | landrace | - |
| 64 | NJAU_C066 | Jiangxi | landrace | III |
| 65 | NJAU_C067 | Shanxi | landrace | II |
| 66 | NJAU_C068 | Guangdong | landrace | III |
| 67 | NJAU_C069 | Guangdong | landrace | III |
| 68 | NJAU_C070 | Guangxi | landrace | III |
| 69 | NJAU_C071 | Huaiyin | Improved | III |
| 70 | NJAU_C072 | Yunnan | landrace | III |
| 71 | NJAU_C073 | Yunhe | landrace | III |
| 72 | NJAU_C074 | Feixi | landrace | III |
| 73 | NJAU_C075 | Jiangxi | landrace | III |
| 74 | NJAU_C076 | Sichuan | landrace | III |
| 75 | NJAU_C077 | Sichuan | landrace | III |
| 76 | NJAU_C078 | Jiangxi | landrace | III |
| 77 | NJAU_C079 | Liaoning | landrace | I |
| 78 | NJAU_C080 | Guangdong | landrace | III |
| 79 | NJAU_C081 | Hunan | landrace | III |
| 80 | NJAU_C082 | Shanghai | landrace | III |
| 81 | NJAU_C083 | Hunan | landrace | III |
| No. | Accession | Origin^a^ | Evolution Type | Soybean ecological regions in China |
| 82 | NJAU_C084 | Gaozhou | landrace | III |
| 83 | NJAU_C085 | Huangbei | landrace | III |
| 84 | NJAU_C086 | Jiangxi | landrace | III |
| 85 | NJAU_C087 | Hunan | landrace | III |
| 86 | NJAU_C088 | Anhui | landrace | II |
| 87 | NJAU_C089 | Hubei | landrace | III |
| 88 | NJAU_C090 | - | landrace | - |
| 89 | NJAU_C091 | Zhejiang | landrace | III |
| 90 | NJAU_C092 | Jiangxi | landrace | III |
| 91 | NJAU_C093 | He'nan | landrace | II |
| 92 | NJAU_C094 | Brazil | landrace |  |
| 93 | NJAU_C095 | Xinchang | landrace | III |
| 94 | NJAU_C096 | Jiangsu | landrace | II |
| 95 | NJAU_C097 | - | - | - |
| 96 | NJAU_C098 | Jiangsu | Improved | III |
| 97 | NJAU_C099 | - | landrace | - |
| 98 | NJAU_C100 | Jiangsu | Improved | - |
| 99 | NJAU_C101 | Beijing | landrace | II |
| 100 | NJAU_C102 | Wenshang | landrace | II |
| 101 | NJAU_C103 | Heilongjiang | Improved | I |
| 102 | NJAU_C104 | Liaoning | Improved | I |
| 103 | NJAU_C105 | Shanghai | landrace | III |
| 104 | NJAU_C106 | Shanxi | Improved | - |
| 105 | NJAU_C107 | Shangqiu | landrace | II |
| 106 | NJAU_C108 | He'nan | landrace | II |
| 107 | NJAU_C109 | He'nan | landrace | II |
| 108 | NJAU_C110 | Hunan | landrace | III |
| 109 | NJAU_C111 | Zhejiang | landrace | III |
| 110 | NJAU_C112 | Fujian | landrace | III |
| 111 | NJAU_C113 | Hubei | landrace | III |
| 112 | NJAU_C114 | Shandong | landrace | II |
| 113 | NJAU_C115 | Liaoning | landrace | I |
| 114 | NJAU_C116 | Jilin | landrace | I |
| 115 | NJAU_C117 | Heilongjiang | landrace | I |
| 116 | NJAU_C118 | Liaoning | landrace | I |
| 117 | NJAU_C119 | Chongqing | landrace | III |
| 118 | NJAU_C120 | - | landrace | - |
| 119 | NJAU_C121 | Jiyuan | landrace | II |
| 120 | NJAU_C123 | Juye | landrace | II |
| 121 | NJAU_C124 | Guizhou | landrace | III |
| 122 | NJAU_C125 | Xuchang | landrace | II |
| No. | Accession | Origin^a^ | Evolution Type | Soybean ecological regions in China |
| 123 | NJAU_C126 | He'nan | landrace | II |
| 124 | NJAU_C127 | He'nan | landrace | II |
| 125 | NJAU_C128 | Shandong | landrace | II |
| 126 | NJAU_C129 | Hebei | landrace | III |
| 127 | NJAU_C130 | Shandong | Improved | II |
| 128 | NJAU_C131 | Liaoning | Improved | I |
| 129 | NJAU_C132 | He'nan | landrace | II |
| 130 | NJAU_C133 | Anhui | landrace | II |
| 131 | NJAU_C135 | - | - | - |
| 132 | NJAU_C137 | Kanagawa | landrace | - |
| 133 | NJAU_C138 | Hubei | Improved | III |
| 134 | NJAU_C139 | Fujian | landrace | III |
| 135 | NJAU_C140 | American | landrace | - |
| 136 | NJAU_C141 | He'nan | landrace | II |
| 137 | NJAU_C142 | Guizhou | landrace | III |
| 138 | NJAU_C143 | Shandong | landrace | II |
| 139 | NJAU_C144 | Nanjing | Improved | III |
| 140 | NJAU_C145 | Guanyun | landrace | II |
| 141 | NJAU_C146 | Jiangsu | landrace | II |
| 142 | NJAU_C147 | Jiangsu | landrace | III |
| 143 | NJAU_C148 | Shandong | landrace | II |
| 144 | NJAU_C149 | Shandong | landrace | II |
| 145 | NJAU_C150 | Guangdong | landrace | III |
| 146 | NJAU_C151 | Shanxi | landrace | - |
| 147 | NJAU_C152 | Taoan | landrace | I |
| 148 | NJAU_C153 | Yixian | landrace | II |
| 149 | NJAU_C154 | Jiangxi | landrace | III |
| 150 | NJAU_C155 | Shandong | landrace | II |
| 151 | NJAU_C156 | Guangxi | landrace | III |
| 152 | NJAU_C157 | Jiangxi | landrace | III |
| 153 | NJAU_C158 | Tong'an | landrace | III |
| 154 | NJAU_C159 | Binhai | landrace | - |
| 155 | NJAU_C160 | Jingde | landrace | III |
| 156 | NJAU_C161 | Fushun | landrace | I |
| 157 | NJAU_C162 | Guizhou | landrace | III |
| 158 | NJAU_C163 | Jiangsu | landrace | III |
| 159 | NJAU_C164 | Shandong | Improved | II |
| 160 | NJAU_C165 | Hubei | Improved | III |
| 161 | NJAU_C166 | Liaoning | Improved | I |
| 162 | NJAU_C167 | Yunnan | landrace | III |
| 163 | NJAU_C168 | He'nan | landrace | II |
| No. | Accession | Origin^a^ | Evolution Type | Soybean ecological regions in China |
| 164 | NJAU_C169 | - | landrace | - |
| 165 | NJAU_C171 | Hubei | landrace | III |
| 166 | NJAU_C172 | Hunan | landrace | III |
| 167 | NJAU_C173 | Hubei | Improved | III |
| 168 | NJAU_C174 | Shanxi | Improved | - |
| 169 | NJAU_C175 | Guizhou | landrace | III |
| 170 | NJAU_C176 | Guizhou | landrace | III |
| 171 | NJAU_C177 | Jiangsu | landrace | II |
| 172 | NJAU_C178 | Wuchang | landrace | III |
| 173 | NJAU_C179 | Jiangxi | landrace | III |
| 174 | NJAU_C180 | Guangxi | landrace | III |
| 175 | NJAU_C181 | Guangxi | landrace | III |
| 176 | NJAU_C182 | Hebei | landrace | - |
| 177 | NJAU_C183 | Sichuan | landrace | III |
| 178 | NJAU_C184 | Jiangxi | landrace | III |
| 179 | NJAU_C185 | Jiangsu | Improved | III |
| 180 | NJAU_C186 | Guizhou | Improved | III |
| 181 | NJAU_C187 | Dapu | landrace | III |
| 182 | NJAU_C188 | Shanxi | landrace | - |
| 183 | NJAU_C189 | Jiangsu | Improved | III |
| 184 | NJAU_C190 | Hubei | Improved | III |
| 185 | NJAU_C191 | Taiwan | landrace | III |
| 186 | NJAU_C193 | He'nan | Improved | II |
| 187 | NJAU_C194 | Jiangsu | landrace | II |
| 188 | NJAU_C195 | He'nan | landrace | II |
| 189 | NJAU_C196 | Shanxi | Improved | - |
| 190 | NJAU_C197 | Sichuan | landrace | III |
| 191 | NJAU_C198 | Hubei | landrace | III |
| 192 | NJAU_C199 | Hubei | landrace | III |
| 193 | NJAU_C200 | - | - | - |
| 194 | NJAU_C201 | American | - | - |
| 195 | NJAU_C202 | Japan | - | - |
| 196 | NJAU_C203 | Anhui | Improved | - |
| 197 | NJAU_C204 | Anhui | landrace | III |
| 198 | NJAU_C205 | Jiangsu | landrace | III |
| 199 | NJAU_C206 | Zhejiang | landrace | III |
| 200 | NJAU_C207 | Illinois | - | - |
| 201 | NJAU_C208 | Jiangsu | landrace | II |
| 202 | NJAU_C209 | Jiangsu | landrace | - |
| 203 | NJAU_C211 | American | - | - |
| 204 | NJAU_C212 | American | - | - |
| No. | Accession | Origin^a^ | Evolution Type | Soybean ecological regions in China |
| 205 | NJAU_C213 | Jiangsu | Improved |  |
| 206 | NJAU_C214 | Shandong | landrace | II |
| 207 | NJAU_C215 | American | - | - |
| 208 | NJAU_C216 | American | - | - |
| 209 | NJAU_C217 | - | landrace | - |
| 210 | NJAU_C218 | Anhui | landrace | - |
| 211 | NJAU_C219 | He'nan | Improved | II |
| 212 | NJAU_C221 | Nanjing | Improved | III |
| 213 | NJAU_C222 | Shijiazhuang | Improved | II |
| 214 | NJAU_C223 | Nanjing | Improved | III |
| 215 | NJAU_C224 | Xiangfan | Improved | III |
| 216 | NJAU_C225 | Nanjing | Improved | III |
| 217 | NJAU_C226 | Nanjing | Improved | III |
| 218 | NJAU_C227 | Nanjing | Improved | III |
| 219 | NJAU_C228 | Qidong | landrace | III |
| 220 | NJAU_C229 | Beijing | Improved | II |
| 221 | NJAU_C230 | Xuzhou | Improved | II |
| 222 | NJAU_C231 | Xuzhou | Improved | II |
| 223 | NJAU_C232 | Zhoukou | Improved | II |
| 224 | NJAU_C233 | Nanjing | Improved | III |
| 225 | NJAU_C234 | Beijing | Improved | II |
| 226 | NJAU_C235 | Nanjing | Improved | III |
| 227 | NJAU_C236 | Nanjing | Improved | III |
| 228 | NJAU_C237 | Nantong | Improved | III |
| 229 | NJAU_C238 | Nanjing | Improved | III |
| 230 | NJAU_C239 | Zhengzhou | Improved | II |
| 231 | NJAU_C240 | Beian | Improved | I |
| 232 | NJAU_C241 | Ji'ning | Improved | II |
| 233 | NJAU_C242 | Guangdong | landrace | III |
| 234 | NJAU_C243 | Nanjing | Improved | III |
| 235 | NJAU_C244 | Guangxi | landrace | III |
| 236 | NJAU_C245 | Jiangxi | landrace | III |
| 237 | NJAU_C246 | Anhui | landrace | III |
| 238 | NJAU_C247 | Fujian | landrace | III |
| 239 | NJAU_C248 | Daan | landrace | I |
| 240 | NJAU_C249 | Guizhou | landrace | III |
| 241 | NJAU_C250 | Zhejiang | landrace | III |
| 242 | NJAU_C251 | Shanxi | landrace | - |
| 243 | NJAU_C253 | Guangdong | landrace | III |
| 244 | NJAU_C254 | Taoan | landrace | I |
| 245 | NJAU_C255 | Huinan | landrace | I |
| No. | Accession | Origin^a^ | Evolution Type | Soybean ecological regions in China |
| 246 | NJAU_C256 | Guanyun | landrace | II |
| 247 | NJAU_C257 | Jimo | landrace | II |
| 248 | NJAU_C258 | Guangxi | landrace | III |
| 249 | NJAU_C259 | Hubei | landrace | III |
| 250 | NJAU_C260 | Nanjing | Improved | III |
| 251 | NJAU_C261 | Shandong | Improved | II |
| 252 | NJAU_C262 | Guangxi | landrace | III |
| 253 | NJAU_C263 | Guizhou | landrace | III |
| 254 | NJAU_C264 | Guizhou | landrace | III |
| 255 | NJAU_C265 | Jiangsu | landrace | II |
| 256 | NJAU_C266 | Shandong | landrace | II |
| 257 | NJAU_C267 | Shandong | landrace | II |
| 258 | NJAU_C268 | He'nan | Improved | II |
| 259 | NJAU_C269 | Jiangsu | Improved | III |
| 260 | NJAU_C270 | - | Improved | - |
| 261 | NJAU_C271 | Nanjing | Improved | III |
| 262 | NJAU_C272 | Nanjing | Improved | III |
| 263 | NJAU_W001 | Linfen | wild | II |
| 264 | NJAU_W002 | Pingdu | wild | II |
| 265 | NJAU_W003 | Yuanqu | wild | II |
| 266 | NJAU_W004 | Neihuang | wild | II |
| 267 | NJAU_W006 | Jiyuan | wild | II |
| 268 | NJAU_W007 | Lishui | wild | III |
| 269 | NJAU_W008 | Jingjiang | wild | III |
| 270 | NJAU_W010 | Beiwuhe | wild | II |
| 271 | NJAU_W011 | Nantong | wild | III |
| 272 | NJAU_W012 | Beihao | wild | II |
| 273 | NJAU_W013 | Puyang | wild | II |
| 274 | NJAU_W015 | Rushan | wild | II |
| 275 | NJAU_W016 | Yunnan | wild | III |
| 276 | NJAU_W017 | Jiangpu | wild | III |
| 277 | NJAU_W019 | Suizhong | wild | I |
| 278 | NJAU_W020 | Gaixian | wild | I |
| 279 | NJAU_W021 | Zhengzhou | wild | II |
| 280 | NJAU_W022 | Anyang | wild | II |
| 281 | NJAU_W023 | Beipiao | wild | I |
| 282 | NJAU_W024 | Beiwuhe | wild | II |
| 283 | NJAU_W025 | Huanglong | wild | II |
| 284 | NJAU_W026 | Beifengshan | wild | II |
| 285 | NJAU_W028 | Duchang | wild | III |
| 286 | NJAU_W029 | Jiuhuashan | wild | III |
| No. | Accession | Origin^a^ | Evolution Type | Soybean ecological regions in China |
| 287 | NJAU_W030 | Chengde | wild | I |
| 288 | NJAU_W031 | - | wild | - |
| 289 | NJAU_W032 | Fusong | wild | I |
| 290 | NJAU_W033 | Jiangpu | wild | III |
| 291 | NJAU_W035 | Changyi | wild | II |
| 292 | NJAU_W036 | Xinjiang | wild | II |
| 293 | NJAU_W037 | Luonan | wild | III |
| 294 | NJAU_W038 | Jiangpu | wild | III |
| 295 | NJAU_W039 | Kezuozhongqi | wild | I |
| 296 | NJAU_W040 | - | wild | - |
| 297 | NJAU_W041 | Yongji | wild | II |
| 298 | NJAU_W042 | Dafeng | wild | III |
| 299 | NJAU_W043 | Yantai | wild | II |
| 300 | NJAU_W044 | Jiuhuashan | wild | III |
| 301 | NJAU_W045 | Jingtai | wild | I |
| 302 | NJAU_W046 | Mouping | wild | II |
| 303 | NJAU_W047 | Lan'gao | wild | III |
| 304 | NJAU_W048 | Wuhe | wild | II |
| 305 | NJAU_W049 | Haicheng | wild | I |
| 306 | NJAU_W050 | Dongfeng | wild | I |
| 307 | NJAU_W051 | Yaoxian | wild | II |
| 308 | NJAU_W052 | Yaoxian | wild | II |
| 309 | NJAU_W055 | Sichuan | wild | III |
| 310 | NJAU_W056 | Shichengxian | wild | III |
| 311 | NJAU_W058 | Wuxian | wild | III |
| 312 | NJAU_W059 | - | wild | - |
| 313 | NJAU_W060 | Lin'an | wild | III |
| 314 | NJAU_W063 | Jiujiang | wild | III |
| 315 | NJAU_W065 | Lishui | wild | III |
| 316 | NJAU_W066 | Suning | wild | II |
| 317 | NJAU_W067 | Zhengning | wild | II |
| 318 | NJAU_W068 | Huanglong | wild | II |
| 319 | NJAU_W069 | Fengxin | wild | III |
| 320 | NJAU_W070 | Liuyang | wild | III |
| 321 | NJAU_W071 | Shennongjia | wild | III |
| 322 | NJAU_W072 | Lvshan | wild | III |
| 323 | NJAU_W073 | Lishu | wild | I |
| 324 | NJAU_W074 | Zhengzhou | wild | II |
| 325 | NJAU_W075 | Xunxian | wild | III |
| 326 | NJAU_W076 | Baofeng | wild | II |
| 327 | NJAU_W077 | Lichuan | wild | III |
| No. | Accession | Origin^a^ | Evolution Type | Soybean ecological regions in China |
| 328 | NJAU_W078 | Chongyang | wild | III |
| 329 | NJAU_W079 | Yanji | wild | I |
| 330 | NJAU_W081 | Zhaluteqi | wild | I |
| 331 | NJAU_W082 | Jiaohe | wild | I |
| 332 | NJAU_W083 | Yongji | wild | I |
| 333 | NJAU_W084 | Ji'an | wild | I |
| 334 | NJAU_W085 | Jianyang | wild | III |
| 335 | NJAU_W086 | Huinan | wild | I |
| 336 | NJAU_W087 | Tieling | wild | I |
| 337 | NJAU_W088 | Huinan | wild | I |
| 338 | NJAU_W089 | Ningguo | wild | III |
| 339 | NJAU_W090 | Fuxin | wild | I |
| 340 | NJAU_W091 | Taihu | wild | III |
| 341 | NJAU_W094 | Hefeng | wild | III |
| 342 | NJAU_W096 | Zixi | wild | III |
| 343 | NJAU_W097 | Liancheng | wild | III |
| 344 | NJAU_W099 | Taining | wild | III |
| 345 | NJAU_W100 | Keyouqianqi | wild | I |
| 346 | NJAU_W101 | Tangyin | wild | II |
| 347 | NJAU_W102 | Tiantai | wild | III |
| 348 | NJAU_W103 | Yangzhou | wild | III |
| 349 | NJAU_W104 | Changbai | wild | I |
| 350 | NJAU_W105 | Yujiang | wild | III |
| 351 | NJAU_W106 | Liangping | wild | III |
| 352 | NJAU_W107 | Changde | wild | III |
| 353 | NJAU_W108 | Quanzhou | wild | III |
| 354 | NJAU_W109 | Guangxi | wild | III |
| 355 | NJAU_W110 | Fuzhou | wild | III |
| 356 | NJAU_W111 | Rongjiang | wild | III |
| 357 | NJAU_W112 | Chengde | wild | I |
| 358 | NJAU_W113 | Heishan | wild | I |
| 359 | NJAU_W114 | Jixi | wild | I |
| 360 | NJAU_W115 | Hailun | wild | I |
| 361 | NJAU_W116 | Huhehaote | wild | I |
| 362 | NJAU_W117 | Pingliang | wild | II |
| 363 | NJAU_W118 | Huangling | wild | II |
| 364 | NJAU_W119 | Liangping | wild | III |
| 365 | NJAU_W120 | Xiangyin | wild | III |
| 366 | NJAU_W121 | Suining | wild | III |
| 367 | NJAU_W122 | Ninglanglaluo | wild | III |

^a^ Name of the places where the accessions from, including country, province, city or county names.

^b^ The symbol “-” means unknown information or information that we are not sure.
